# Stochastic modeling of economic risk and net return distributions for feedlot steers marketed at alternative endpoints

**DOI:** 10.1093/jas/skaf074

**Published:** 2025-03-10

**Authors:** Lucas M Horton, Ted C Schroeder, Marshall N Streeter, John P Hutcheson, David G Renter

**Affiliations:** Center for Outcomes Research and Epidemiology, Department of Diagnostic Medicine and Pathobiology, College of Veterinary Medicine, Kansas State University, Manhattan, KS, USA; Department of Agricultural Economics, Kansas State University, Manhattan, KS, USA; Merck Animal Health, Lenexa, KS, USA; Merck Animal Health, Lenexa, KS, USA; Center for Outcomes Research and Epidemiology, Department of Diagnostic Medicine and Pathobiology, College of Veterinary Medicine, Kansas State University, Manhattan, KS, USA

**Keywords:** beef, days-on-feed, economics, endpoint management, feedlot, net return, steers

## Abstract

Feedlot cattle in the United States have been progressively fed to heavier, more extreme endpoints (**EPs**) in recent decades. The primary objective was to evaluate economic risk associated with extending days-on-feed (**DOF**) by characterizing net return distributions of feedlot steers fed to later EPs, compared to current industry standards. Stochastic simulation modeling was employed to simulate a variety of conditions, including cattle performance, health, carcass characteristics, and economic market variability at the pen-level. The model was parameterized using data from a large commercial feedlot trial and industry reports. The trial involved cattle harvested at four EPs (EP1, EP2, EP3, EP4), each separated by 14 DOF. EP1 represented the industry standard, with live and carcass weights reflective of industry averages from 2021 to mid-2024. The model’s final outcome was net return difference—the difference in net returns for EP2, EP3, and EP4 compared to if pens had alternatively been marketed at EP1 under a given set of simulated conditions. Conditional random forest models were used to compute variable importance scores to determine the most influential factors on net return differences. Results indicated that as steers were fed to later EPs, net return distributions widened, reflecting increased economic risk. Steers marketed on a dressed (carcass) basis using a grid for premiums and discounts showed a higher frequency of negative net returns compared to a live marketing basis, primarily due to discounts for Yield Grade and heavyweight carcasses. Across both sale basis, negative net returns became more frequent with increasing EPs. The most influential variable was the difference in fed cattle prices received between later-fed EPs and EP1, accounting for price changes when delaying marketing. Other important economic factors included base-fed cattle prices, corn prices, and the Quality Grade grid when grid marketing. Mortality risk was the most important noneconomic variable, while other animal performance variables, such as weight and carcass traits, were of marginal to minimal importance. These findings highlight the dominant role of economic factors on net returns when feeding steers to later EPs, emphasizing the need to prioritize market conditions in EP management decisions.

## Teaser

Economic uncertainty and variability increase when feeding steers beyond industry standards, and the economic endpoint providing the highest expected net returns may not align with perceived ideal animal endpoints due to the importance of fluctuating market conditions.

## Introduction

Over the past several decades, feedlot cattle in the United States have been fed to increasingly heavier endpoints (Livestock Marketing Information Center; LMIC). Although the beef-cow herd size has declined over the last 50-plus years, total beef production has continued to rise due to advancements in genetics, technologies, and management practices (Wileman and Thomson, 2010; Meyer and Gunn, 2015). Numerous research efforts have evaluated the effects of extending days-on-feed (**DOF**) on the performance and carcass characteristics of feedlot cattle, providing guidance on endpoint (**EP**) management. Much of this work has been summarized by Galyean et al. (2023). As feedlot cattle are fed longer, they accumulate more total weight and fat deposition, leading to higher United States Department of Agriculture (**USDA**) Quality and Yield Grades (**QG** and **YG**, respectively). However, live performance metrics such as daily gain and feed efficiency decline as DOF increases. This trend can be misleading, as the rate of carcass weight gain may increase while live weight gain rates decrease (Wilken et al., 2015). This is because carcass weight gain as a proportion of live weight gain increases as steers and heifers are fed for longer periods. This concept, known as carcass transfer or incremental dressing percentage, has been documented by several studies (Streeter et al., 2012; Walter et al., 2018; Ohnoutka et al., 2021; Horton et al., 2023). Thus, when extending the DOF of feedlot cattle, it may be advantageous to market them on a dressed (carcass) basis rather than by live weight (Wilken et al., 2015; Horton et al., 2024), due in part to incremental dressing percentage.

Although extensive research has been conducted on differing DOF and feedlot cattle EPs, concurrent economic analyses have been relatively scarce despite economics being a major consideration for producers. It has been suggested that profitability may increase by feeding steers beyond industry norms (Wilken et al., 2015; Poss et al., 2022). It is also important to consider that the cost of underfeeding cattle may be lower than the cost of overfeeding beyond an “optimal” EP (Koontz et al., 2008). However, current industry weights now surpass those referenced in these studies, reflecting shifts in industry standards. In a sensitivity analysis of economic factors, Horton et al. (2024) found minimal evidence supporting feeding beef heifers beyond a baseline EP. Their study demonstrated that net return differences are influenced by variable market conditions. Horton et al. (2024) characterized the relative impact of individual pricing variables on net returns while holding other factors constant. While this approach is valuable for isolating effects of individual variables, economic factors, and cattle populations fluctuate concurrently. As a result, the potential range of outcomes is broader than those described by Horton et al. (2024), with different combinations of market conditions and cattle characteristics potentially leading to varying optimal EPs for feedlot cattle.

An important consideration in extending DOF is added risk. Economic risk includes inherent uncertainty of variable market conditions (Rushton, 2007). Various methods of analysis can account for economic risk, with stochastic simulation being a widely accepted strategy for modeling and characterizing such uncertainty (Corbin and Griffin, 2006; Rushton, 2007). Stochastic simulation involves specifying variables of interest within probability distributions, accounting for correlations between and within variables, and simulating numerous iterations—often thousands—to generate distributions of potential outcomes under varying conditions (Corbin and Griffin, 2006). This approach has been applied to address a variety of economic issues in animal agriculture (Belasco et al., 2009; Silva et al., 2017; Dennis et al., 2020).

A substantive task in stochastic simulation is deciding what to model as stochastic and parameterizing the model. Recently, Martinez et al. (2023) conducted a robust randomized controlled trial evaluating effects of feeding steers beyond industry-standard EPs. Using trial data combined with other industry data to parameterize a stochastic simulation model enables the characterization of economic risk when feeding steers to later EPs under recent market conditions. This information is valuable for producers and industry stakeholders, as it provides expected variability of net returns when feeding to alternative EPs. Therefore, the primary objective of this study was to characterize and evaluate marginal net return distributions for beef feedlot steers fed to later EPs by using stochastic simulation of key economic and performance variables, with cattle marketed either live or dressed with a carcass-based premium and discount grid. A secondary objective was to assess the relative importance of economic and cattle performance variables on net return variation when feeding to later EPs and to describe the conditions of these variables when extended feeding was unfavorable or advantageous.

## Materials and methods

### Background information

A combination of industry-reported data and trial data (Martinez et al., 2023) were used for parameterization of a stochastic cattle feeding net return simulation model. The trial employed a 3 × 4 factorial design with implant program (**IP**) and DOF as factors in a randomized complete block design. Three IP treatments were compared: a single extended-release implant at initial processing and two reimplant programs with differing timing of terminal implant delivery. Treatments in the DOF factor were steers harvested at 166, 180, 194, or 208 DOF. A total of 6,840 crossbred beef steers were blocked by arrival, and pens were randomly assigned to treatment within block. Blocks were allocated over three enrollment periods spanning different seasons: fall 2019, late spring 2020, and late winter to early spring 2021. The trial was conducted at a commercial feedlot in the Texas Panhandle. For further detail (e.g., dietary formulations, cattle management), the reader is referred to Martinez et al. (2023).

For the simulation model, most published results from Martinez et al. (2023) could not be used for parameterization as we required either different or alternative forms of the outcomes. Raw data were obtained for the analyses, hereafter referred to as “trial data.” We reclassified the DOF factor as “endpoint” (EP), since actual DOF is relative to initial body weight (**BW**). We evaluated four feedlot steer EPs which are characterized by final weight and carcass composition, each separated by 14 DOF. Therefore, the four DOF treatments were classified as Endpoint 1, Endpoint 2, Endpoint 3, and Endpoint 4 (EP1, EP2, EP3, and EP4, respectively). The simulation model framework considered only events occurring after EP1, as prior conditions are irrelevant for assessing the incremental economic impacts of extending DOF. Analysis of the trial data (described in a later section) showed no significant IP × EP interactions for outcomes required for parameterization. Therefore, only main effect estimates of EP were used for parameterization, as there was no evidence that EP estimates depended on IP treatment.

### Simulation model framework

Each individual simulation (i.e., iteration) compared four scenarios representing the same pen of feedlot steers marketed at each of the four EPs. The key question was: *what* would have been the marginal net return difference *if* we had marketed the same pen at each EP? Although producers ultimately need to decide on a single marketing date (EP) in advance, and what would have happened to net returns at alternative EPs is largely unknown, by framing the simulations in this manner, we can better inform producers or other decision-makers by providing net return expectations when marketing at these alternative EPs. In essence, each iteration simulated net returns for the same pen, *if* it had been fed to EP1, *if* it had been fed to EP2, *if* it had been fed to EP3, or *if* it had been fed to EP4, ultimately computing marginal net return differences for EP2, EP3, and EP4 compared to EP1. Endpoint 1 represents the contemporary industry standard based on recent finished weights; thus, marketing earlier than EP1 was not considered. Since our interest is in the marginal net return differences compared to EP1, only gross revenue is simulated for EP1, as prior costs (e.g., feeder cattle purchase, feed cost, health events) are “sunk” and constant across EPs, and would not impact net return differences. For EP2, EP3, and EP4, health risks (e.g., morbidity and mortality) and costs associated with longer feeding were incorporated, along with revenue. All simulations were conducted at the pen-level, with EP1 having 200 steers marketed. We considered two primary sale bases: which were *if* the steers were marketed on a live basis, or *if* the steers were marketed dressed (i.e., carcass) basis with a premium and discount grid (termed “grid sale basis”). Marketing on a dressed-cash basis without a grid was also evaluated, but as relatively few cattle are sold in this manner in the United States, results are confined to supplementary material and are minimally discussed.

The simulation model was stochastic, with random values drawn from specified probability distributions for each simulated economic and cattle variable (health, performance, carcass characteristics). Furthermore, correlation matrices were used to account for relationships between and within certain simulated variables. Some variables were held constant across simulations, varied in a sensitivity analysis, or predicted based on estimated regression relationships with a simulated variable.

In each iteration, EP1 serves as a baseline with simulated characteristics (e.g., weight, distribution of QG and YG) that are then built upon correspondingly for later EPs using correlation matrices or simulated coefficients (e.g., average daily gain). Events occurring between EPs (e.g., mortalities) were carried over to subsequent EPs, linking them together. Revenue was calculated for all EPs by multiplying cattle variables by corresponding pricing variables. For EP2, EP3, and EP4, added production costs and costs from adverse health events were subtracted to obtain net return post-extended DOF. Marginal pen-level net return differences compared to EP1 were computed by subtracting EP2, EP3, and EP4 returns from EP1 revenue. This partial budgeting approach measures the dollar gain or loss when feeding the pen beyond EP1, without estimating overall profitability since costs prior to EP1 are not included. For interpretation, pen-level net return differences are converted to net return difference per animal marketed. With the simulation model framework described, components are detailed in the following sections.

### Statistical analyses of simulation model variables

Variables from the trial data used to parameterize the simulation model were validated, and their estimates and variability were extracted. Validation involved 1) evaluating potential IP × EP interactions, 2) testing for treatment effects and linear and quadratic effects of EP, and 3) generating parameter estimates for use in the simulation model. General and generalized linear mixed models (**LMM** and **GLMM**, respectively) were employed (Proc GLIMMIX, SAS 9.4; SAS Institute Inc., Cary, NC), with pen as the experimental unit. To assess validations 1) and 2), IP, EP, and the IP × EP interaction were modeled as fixed effects, with random intercepts for period (*n* = 3 enrollment periods) and block within period to account for clustering. Linear and quadratic orthogonal polynomial contrasts for EP were included. Continuous outcomes (i.e., dry matter intake [**DMI**], final BW, and hot carcass weight [**HCW**]) were modeled using LMM with a Gaussian distribution, identity link function, Kenward-Roger degrees of freedom adjustment, and restricted maximum likelihood estimation. Model assumptions of normality and homogeneous residual variances were assessed visually using plots of conditional and marginal studentized residuals. Ordinal outcomes (i.e., QG and YG) were modeled using GLMM with a multinomial distribution, cumulative logit link function, Kenward-Roger degrees of freedom adjustment, and residual pseudo-likelihood estimation. Since these ordinal data were at the individual carcass-level (unlike pen-level for other outcomes), an additional random intercept for pen was included to account for clustering of steers within pens. Significance was declared a priori at α = 0.05.

There were no IP × EP interactions (*P*-values ≥ 0.27), indicating that incorporation of interactive effects into the simulation model was not needed. There also was no evidence of quadratic effects with increasing EPs (*P*-values ≥ 0.43). All outcomes had significant main (*P*-values < 0.01) and linear effects (*P*-values < 0.01) for EP, except for DMI, which had no significant EP (*P* = 0.29) or linear effect (*P* = 0.35). Notably, the DMI analyzed was for the final 14 DOF for each EP only (i.e., consumption prior to EP1 is irrelevant), as we tested whether differences in DMI between EPs existed during their final 14 DOF (corresponding to the 14 d separating each EP). The results indicate no evidence to suggest a change in mean DMI as DOF increased.

### Conceptual model and parameterization

For each pen *i* (with *i* = 1,..., *N* simulations), the framework of variables that were simulated ($\tilde{X}$), varied for sensitivity analysis ($\overset{\cdots }{X}$), fixed, and subsequent calculations with relevant expressions are outlined herein. To frame this assessment under contemporary cattle populations and pricing conditions, reported industry data were sourced from January 1, 2021 to June 16, 2024. Animal performance, health, and carcass variables were simulated, except for the percentage of heavyweight carcasses, which was derived from the simulated HCW (Table 1). To establish baseline final BW and HCW for EP1 in each iteration (the *i*^*th*^ pen), we calculated the approximate mean (667 kg final BW, 431 kg HCW; rounded up) and standard deviation from the “5 Area Weekly Weighted Average Direct Slaughter Cattle – Negotiated Purchases” report (LMIC; USDA Agricultural Marketing Service [**AMS**] data). These baseline weights and their variability reflect contemporary reported weights for steers marketed at EP1 and are nearly identical to EP1 weights in the trial data (Martinez et al., 2023). Final live BW (${\tilde{FBW}}_{i,EP1}$) and HCW (${\tilde{HCW}}_{i,EP1}$) were first simulated for pen *i* at EP1, drawing from a normal distribution (Table 1), and were correlated (using a 2 × 2 matrix) so that within each iteration, final BW and HCW corresponded realistically. Instead of simulating the percentage of heavyweight carcasses (weights >476 kg [1,050 lb]), this was calculated by assuming a normal distribution of individual HCWs (with SD 38.6 kg) around the mean simulated HCW for each EP and determining the percentage exceeding the heavyweight threshold (Table 1). The same SD derived from the trial data of within-pen individual carcasses was used across all simulations. The probability or proportion of heavyweight carcasses ($Heavy_{i,EP1}$) was calculated, where *Φ*(·) is the standard normal cumulative distribution function, with a constant within-pen SD (σ), and may be expressed as:

**Table 1.** Parameterization of simulated economic, cattle performance, carcass, and health variables used for evaluation of net return differences when feeding beef steers to later endpoints

Variable^1^	Mean	SD	Distribution	Source
Corn price, $/bu	6.00	1.10	Log-normal	LMIC
Live fed cattle price, $/cwt	152.00	25.10	Log-normal	LMIC
Dry matter intake, kg/d	10.5	0.58	Normal	Trial data
EP1 final live weight, kg	667	11.4	Normal	LMIC
EP1 hot carcass weight, kg	431	8.8	Normal	LMIC
Live weight gain, kg/d	1.32	0.06	Normal	Trial data
Carcass weight gain, kg/d	1.00	0.04	Normal	Trial data
Percent heavyweight carcasses,^2^ kg	–	38.6	–	Trial data
Mortality, n/1,000 animal-days	0.09	–	Poisson	Trial data
Removal, n/1,000 animal-days	0.18	–	Poisson	Trial data
Morbidity, n/1,000 animal-days	0.29	–	Poisson	Trial data
Ordinal variables	Mean	Scale	Distribution	Source
EP1 Yield Grade distribution, log odds			Logistic	Trial data
5	−3.59	0.170		
4	−1.72	0.167		
3	0.15	0.166		
2	2.29	0.170		
EP1 Yield Grade coefficient, log odds			Logistic	Trial data
EP2	0.43	0.081		
EP3	0.73	0.081		
EP4	1.31	0.081		
EP1 Quality Grade distribution, log odds			Logistic	Trial data
Prime	−3.67	0.332		
Choice	0.82	0.322		
Select	4.62	0.362		
Quality Grade coefficient, log odds			Logistic	Trial data
EP2	0.31	0.067		
EP3	0.52	0.067		
EP4	0.73	0.067		

^1^Abbreviations: bu = bushel (25.4 kg or 56 lb); cwt = hundred-weight (45.4 kg or 100 lb); EP1 = endpoint 1.

^2^Heavyweight carcasses were not directly simulated, rather they were estimated by assuming the specified SD around the mean simulated hot carcass weight and calculating the percent that would be over 476 kg (1,050 lb).


$$\begin{array}{l} Heavy_{i,EP1}=1-\;\Phi \left(\frac{476\;kg-\;{\tilde{HCW}}_{i,EP1}}{\sigma_{38.6\;kg}}\right) \end{array}$$

Simulation of carcass YG and QG for each EP was based on multinomial GLMM analyses of the trial data, using a proportional odds framework (Dohoo et al., 2009). In this framework, the model estimated separate intercepts (cumulative log odds) for each grade, representing the probability of being in that grade or higher for EP1 (the referent EP). Referent grades were YG 1 and sub-Select, and their corresponding intercepts were dropped. This setup allowed establishment of a baseline distribution of YGs and QGs for EP1, from which subsequent EP distributions could be adjusted. Mean cumulative log odds estimates for EP1 are provided in Table 1. Scale parameters in the logistic simulations controlled the spread of the distributions (i.e., the amount of variability to simulate), and were parameterized to produce 2.5^th^ and 97.5^th^ percentile log odds matching the 95% confidence interval (**CI**) of intercepts and coefficients from the GLMM estimates. For each pen *i*, the baseline YG distribution was simulated on the cumulative logit (L) scale using a series of correlated logistic regressions (a 4 × 4 matrix derived from the GLMMs) constructed in the proportional odds model framework. The correlation matrix for YG (and later QG) accounted for relationships between grades in the EP1 baseline distribution, ensuring that grades closer together were more correlated, preserving biological realism of the distributions. Let


$$\begin{array}{l} L{\tilde{(YG}}_{i,EP1})=\;\left[L{\tilde{(YG}}_{i,EP1}^{\left(5\right)}),\;L{\tilde{(YG}}_{i,EP1}^{\left(4\right)}),\;L\left({\tilde{YG}}_{i,EP1}^{\left(3\right)}\right),\;L\left({\tilde{YG}}_{i,EP1}^{\left(2\right)}\right)\;\right] \end{array}$$

be the vector of cumulative logit values for the thresholds corresponding to the five YG categories. Note that one threshold (YG 1) is dropped as the referent as its cumulative probability is set to 1, so there are four latent cumulative logits. These cumulative logits are then transformed into cumulative probabilities (the probability of being a given grade or higher) using the logistic function:


$$\begin{array}{l} f\left\{L\right\}=\;\frac{1}{1+exp\left(-L\right)} \end{array}$$

Then, individual probabilities for each grade were calculated by subtracting the probability of being in the next higher grade, where YG proportions for pen *i* are obtained by differencing:


$$\begin{array}{l} {\tilde{YG}}_{i,EP1}^{\left(5\right)}=f\left\{L\left({\tilde{YG}}_{i,EP1}^{\left(5\right)}\right)\right\}, \end{array}$$

$$\begin{array}{l} {\tilde{YG}}_{i,EP1}^{\left(4\right)}=f\left\{L\left({\tilde{YG}}_{i,EP1}^{\left(4\right)}\right)\right\}-f\left\{L\left({\tilde{YG}}_{i,EP1}^{\left(5\right)}\right)\right\}, \end{array}$$

$$\begin{array}{l} {\tilde{YG}}_{i,EP1}^{\left(3\right)}=f\left\{L\left({\tilde{YG}}_{i,EP1}^{\left(3\right)}\right)\right\}-f\left\{L\left({\tilde{YG}}_{i,EP1}^{\left(4\right)}\right)\right\}, \end{array}$$

$$\begin{array}{l} {\tilde{YG}}_{i,EP1}^{\left(2\right)}=f\left\{L\left({\tilde{YG}}_{i,EP1}^{\left(2\right)}\right)\right\}-f\left\{L\left({\tilde{YG}}_{i,EP1}^{\left(3\right)}\right)\right\}, \end{array}$$

$$\begin{array}{l} {\tilde{YG}}_{i,EP1}^{\left(1\right)}=1-f\left\{L\left({\tilde{YG}}_{i,EP1}^{\left(2\right)}\right)\right\} \end{array}$$

This produces a vector of within-pen YG proportions, where proportions of the *j*^*th*^ grade equal 1 when summed:


$$\begin{array}{l} {\tilde{YG}}_{i,EP1}=\;\left[{\tilde{YG}}_{i,EP1}^{\left(5\right)},\;{\tilde{YG}}_{i,EP1}^{\left(4\right)},\;{\tilde{YG}}_{i,EP1}^{\left(3\right)},\;{\tilde{YG}}_{i,EP1}^{\left(2\right)},\;{\tilde{YG}}_{i,EP1}^{\left(1\right)}\;\right],\;with\;\underset{j=1}{\overset{5}{\sum }}\;{\tilde{YG}}_{i,EP1}^{\left(\;j\right)}=1 \end{array}$$

The transformed proportions for EP1 YGs resulted in median values of 2.7%, 12.5%, 38.5%, 37.2%, and 9.2% for YG 5, 4, 3, 2, and 1, respectively.

Simulations of QG used the same process as described for YG. For notation purposes, Prime, Choice, Select, and sub-Select grades are indicated by numbers 4, 3, 2, and 1, respectively. Mean cumulative log odds estimates and scale parameters for EP1 QG distributions are provided in Table 1. Endpoint 1 QG simulations were again correlated, which used a 3 × 3 matrix derived from the GLMMs. Let


$$\begin{array}{l} L{\tilde{(QG}}_{i,EP1})=\;\left[\;L{\tilde{(QG}}_{i,EP1}^{\left(4\right)}),\;L\left({\tilde{QG}}_{i,EP1}^{\left(3\right)}\right),\;L\left({\tilde{QG}}_{i,EP1}^{\left(2\right)}\right)\;\right] \end{array}$$

be the vector of cumulative logit values for the thresholds corresponding to the four QG categories. Sub-Select was dropped as the referent QG. These cumulative logits were then transformed into cumulative probabilities using the previously described logistic function, *f*{·}. Endpoint 1 QG proportions were again calculated by differencing:


$$\begin{array}{l} {\tilde{QG}}_{i,EP1}^{\left(4\right)}=f\left\{L\left({\tilde{QG}}_{i,EP1}^{\left(4\right)}\right)\right\}, \end{array}$$

$$\begin{array}{l} {\tilde{QG}}_{i,EP1}^{\left(3\right)}=f\left\{L\left({\tilde{QG}}_{i,EP1}^{\left(3\right)}\right)\right\}-f\left\{L\left({\tilde{QG}}_{i,EP1}^{\left(4\right)}\right)\right\}, \end{array}$$

$$\begin{array}{l} {\tilde{QG}}_{i,EP1}^{\left(2\right)}=f\left\{L\left({\tilde{QG}}_{i,EP1}^{\left(2\right)}\right)\right\}-f\left\{L\left({\tilde{QG}}_{i,EP1}^{\left(3\right)}\right)\right\}, \end{array}$$

$$\begin{array}{l} {\tilde{QG}}_{i,EP1}^{\left(1\right)}=1-f\left\{L\left({\tilde{QG}}_{i,EP1}^{\left(2\right)}\right)\right\} \end{array}$$

This results in a within-pen vector of QG proportions, where proportions of the *j*^*th*^ grade equal 1 when summed:


$$\begin{array}{l} {\tilde{QG}}_{i,EP1}=\;\left[{\tilde{QG}}_{i,EP1}^{\left(4\right)},\;{\tilde{QG}}_{i,EP1}^{\left(3\right)},\;{\tilde{QG}}_{i,EP1}^{\left(2\right)},\;{\tilde{QG}}_{i,EP1}^{\left(1\right)}\;\right],\;with\;\underset{j=1}{\overset{4}{\sum }}\;{\tilde{QG}}_{i,EP1}^{\left(\;j\right)}=1 \end{array}$$

The transformed proportions for EP1 QGs resulted in median values of 2.5%, 66.9%, 29.6%, and 1.0% for Prime, Choice, Select, and sub-Select grades, respectively.

After simulating baseline characteristics for EP1 pens, performance, carcass, and health changes for later-fed EPs could be accounted for. For notation purposes, let EPι (ι ∈{2, 3, 4}) represent the later-fed EPs. We simulated the rate of gain on both live and carcass weight bases to calculate finished weights for EPι when extending DOF. To determine appropriate rates of gain, LMMs for final BW and HCW were modeled as described in the statistical analysis of simulation model variables section, except EP was modeled as a continuous variable for DOF rather than categorical. This resulted in adjusted coefficients expressed as weight gain per day (1.32 kg/day live BW gain, 1.00 kg/day HCW gain). This approach assumes linearity with increasing EP, which was supported by our trial data analysis. The weight gain coefficients were simulated from a normal distribution, with SDs specified to produce 2.5^th^ and 97.5^th^ percentile values matching the 95% CI around the mean weight gain coefficients from the LMMs (Table 1). These coefficients were simulated (and correlated in a 2 × 2 matrix, similar to EP1 final BW and HCW) and multiplied by the additional DOF required for each EPι to determine total weight gained. Within each iteration, the same simulated coefficients for weight gain were used across EPι to calculate their mean final live BW and HCW. This process can be expressed as:


$$\begin{array}{l} {\tilde{FBW}}_{i,EP\iota }=\;{\tilde{FBW}}_{i,EP1}+\;{\tilde{LDG}}_{i}\times DOF_{EP\iota } \end{array}$$

$$\begin{array}{l} {\tilde{HCW}}_{i,EP\iota }=\;{\tilde{HCW}}_{i,EP1}+\;{\tilde{CDG}}_{i}\times DOF_{EP\iota } \end{array}$$

where ${\tilde{LDG}}_{i}$ is live daily gain and ${\tilde{CDG}}_{i}\;$ is carcass daily gain per steer in pen *i*, and $DOF_{EP\iota }$ is the additional days fed for each EPι (14, 28, or 42). The probability or proportion of heavyweight carcasses for the *i^th^* pen at EPι was derived from ${\tilde{HCW}}_{i,EP\iota }$ using the same procedure as previously described for EP1, where:


$$\begin{array}{l} Heavy_{i,EP\iota }=1-\;\Phi \left(\frac{476\;kg-\;{\tilde{HCW}}_{i,EP\iota }}{\sigma_{38.6\;kg}}\right) \end{array}$$

Distributions of EP1 carcass grades were shifted upward for EPι, accounting for the effects of adding DOF. For each EPι, the cumulative log odds for YG and QG were adjusted by adding coefficients derived from the GLMMs to the baseline EP1 coefficients (i.e., intercepts), reflecting the proportional odds assumption (Dohoo et al., 2009). The log odds coefficients for EPι YG and QG adjustments and the scale parameters used for simulation (logistic distribution) are in Table 1. Log odds coefficients (β) for adjusting the baseline distribution for EPι were generated with correlated simulations (from a 3 × 3 matrix) to ensure consistent proportional changes between EPs within each iteration. Unlike weight gain coefficients that were multiplied by $DOF_{EP\iota }$, the βs here implicitly include the additional DOF for each EPι. Baseline EP1 latent logit (L) adjustments are made for EPι, where βYG is the simulated adjustment for the *i*^*th*^ pen at EPι, resulting in YG distribution shifts for extending DOF:


$$\begin{array}{l} L\left({\tilde{YG}}_{i,EP\iota }\right)=L\left({\tilde{YG}}_{i,EP1}\right)\;+\;{\tilde{\beta YG}}_{i,EP\iota } \end{array}$$

The adjusted latent vector is then back-transformed via the same logistic function for cumulative probabilities as previously described (*f*{·}), followed by the same differencing procedure used for EP1 calculation of individual within-pen YG proportions (not shown here), resulting in a vector of YG for EPι.


$$\begin{array}{l} {\tilde{YG}}_{i,EP\iota }=f\left\{L\left({\tilde{YG}}_{i,EP\iota }\right)\right\},\;with\;\underset{j=1}{\overset{5}{\sum }}\;{\tilde{YG}}_{i,EP\iota }^{\left(\;j\right)}=1 \end{array}$$

The same process was followed for EPι QG adjustments, where the adjustment coefficient (βQG) is simulated for the *i*^*th*^ pen and applied to the logit cumulative probabilities of EP1:


$$\begin{array}{l} L\left({\tilde{QG}}_{i,EP\iota }\right)=L\left({\tilde{QG}}_{i,EP1}\right)\;+\;{\tilde{\beta QG}}_{i,EP\iota } \end{array}$$

Back-transformation and differencing procedures were executed as previously described, resulting in a vector of within-pen QG proportions for EPι:


$$\begin{array}{l} {\tilde{QG}}_{i,EP\iota }=f\left\{L\left({\tilde{QG}}_{i,EP\iota }\right)\right\},\;with\;\underset{j=1}{\overset{4}{\sum }}\;{\tilde{QG}}_{i,EP\iota }^{\left(\;j\right)}=1 \end{array}$$

We assumed that risk of health events occurring before harvest increases with additional DOF. Although most health events in feedlots occur early in the feeding period (Edwards, 1996; Sanderson et al., 2008; Babcock et al., 2009), the risk during extended feeding was expected to be small, but potentially costly. In addition to morbidity and mortality health outcomes, “removals” are cattle culled from the pen for health reasons (i.e., railers) and sold separately at a discounted price. To parameterize health outcomes, additional GLMMs were modeled using the trial data. Health data for EP1 pens were excluded, and only events occurring *after* EP1 harvest (within block) for EPι pens were included. These pen-level count data were analyzed with random-effects-only GLMMs using a Poisson distribution and log link function, with block within season as a random intercept and log animal-days-at-risk as the offset. The models first employed a Laplace approximation to evaluate overdispersion; in the absence of overdispersion, they were re-fit using residual pseudo-likelihood estimation. The intercept (log incidence rate ratio) was back-transformed to obtain the model-adjusted mean incidence per animal-day-at-risk. Mean incidence rate estimates for morbidity, removal, and mortality were multiplied by 1,000 for interpretation and are expressed in Table 1 as events per 1,000 animal-days-at-risk. Note that for pens where no simulated fallouts (removal or mortality) occur, there would be 2,800, 5,600, and 8,400 animal-days-at-risk for EP2, EP3, and EP4, respectively.

Potential morbidity, mortality, and removals (*Morb*, *Mort*, and *Rem*, respectively) were simulated for EPι, accounting for additional days-at-risk for health events to occur. For each pen *i*, the expected animal-days (denoted *ExpDays* in expressions) that occur between EP intervals are calculated and used for the simulation of health events. Let: *Hd*_*i,EP(ι-1)*_ be the animal (“head”) count at the beginning of the interval (for ι = 2, *Hd*_*i,EP(ι-1)*_ = *Hd*_*i,EP1*_* = *200); and the expected animal‐days for the interval from EP(ι − 1) to EPι where DOF = 14 (i.e., the exposure) are defined as:


$$\begin{array}{l} ExpDays_{i,EP\iota }=\;Hd_{i,EP\left(\iota -1\right)}\;\times DOF \end{array}$$

Health events for the intervals were simulated as Poisson random variables using these effective animal‐days, where λ is the mean incidence per animal-day derived from the trial data (Table 1). Since in a Poisson distribution the variance equals the mean, no separate measure of variability is used. ${\tilde{Morb}}_{i,EP\iota }$, ${\tilde{Mort}}_{i,EP\iota }$, and ${\tilde{Rem}}_{i,EP\iota }$ are the simulated numbers of morbidities, mortalities, and removals for pen *i* during the interval.


$$\begin{array}{l} {\tilde{Morb}}_{i,EP\iota }\;∼\;\lambda_{Morb}\;\times \;ExpDays_{i,EP\iota } \end{array}$$

$$\begin{array}{l} {\tilde{Mort}}_{i,EP\iota }\;∼\;\lambda_{Mort}\;\times \;ExpDays_{i,EP\iota } \end{array}$$

$$\begin{array}{l} {\tilde{Rem}}_{i,EP\iota }\;∼\;\lambda_{Rem}\;\times \;ExpDays_{i,EP\iota } \end{array}$$

Health events occurring between intervals were carried over to the next EP. For example, if two removals occurred between EP1 and EP2, those removals would also be present at EP3, *plus* any additional removals occurring between EP2 and EP3, and so on. Also, revenues from removals in earlier EPs carried over to subsequent EPs. Fallouts reduced the number of expected animal-days-at-risk for subsequent EPs, as there were fewer animals to experience health events. This procedure can be outlined as follows: after simulating ${\tilde{Mort}}_{i,EP\iota }$, and ${\tilde{Rem}}_{i,EP\iota }$ for a given interval, the animal count for the next interval for pen *i* is updated prior to simulation and can be expressed as:


$$\begin{array}{l} Hd_{i,EP\iota }=\;Hd_{i,EP\left(\iota -1\right)}-\;{\tilde{Mort}}_{i,EP\iota }-\;{\tilde{Rem}}_{i,EP\iota } \end{array}$$

This update implicitly carries over the events from previous intervals (i.e., cumulative incidence up to EPι), e.g., health events that occur for a given EPι are carried over and counted along with any events that occur for the subsequent EPι. Although morbidity events do not reduce animal counts and are therefore not included in the prior expression, they similarly accumulated (were summed across EPι). For each health event (*h* ∈{*Morb*, *Mort*, *Rem*}), this could alternatively be expressed as:


$$\begin{array}{l} Health_{h,i,EP\iota }^{total}=\;Health_{h,i,EP\left(\iota -1\right)}^{total}+\;Health_{h,i,EP\iota },\;where\;Health_{h,i,EP1}^{total}=0\; \end{array}$$

Following the simulation of health events, total animal-days (*HdDays*) could be calculated, which was required for calculations of pen-level DMI. Since the Poisson simulations did not specify when health events occurred within the interval, we assumed animal fallouts occurred midway between EPs. The effective animal-days for the interval are given by:


$$\begin{array}{l} HdDays_{i,EP\iota }=\;Hd_{i,EP\left(\iota -1\right)}\;\times DOF-\left({\tilde{Mort}}_{i,EP\iota }+\;{\tilde{Rem}}_{i,EP\iota }\right)\;\times \;\frac{DOF}{2} \end{array}$$

The rationale is that animals remaining throughout the interval contribute 14 DOF, whereas those removed are assumed to exit, on average, midway (i.e., contributing 14/2 d). Finally, the cumulative (or total) animal‐days up to EPι is given by:


$$\begin{array}{l} HdDays_{i,EP\iota }^{total}=\;\underset{\iota^{\prime }=2}{\overset{\iota }{\sum }}\;HdDays_{i,EP\iota^{\prime }} \end{array}$$ .

DMI was simulated using the mean DMI (10.5 kg/d), and SD averaged across all EPs over their final 14 DOF in the trial data (Table 1). As previously noted, there was no evidence of DMI changing with increasing DOF in the trial data; therefore, the same simulated DMI was used for EPι within each simulation iteration. Mean DMI per animal was simulated for pen *i* (${\tilde{DMI}}_{i}$), drawing from a normal distribution, and total pen-level DMI for EPι was derived from this simulation when multiplied by the number of animal-days for the interval, which is given as:


$$\begin{array}{l} DMI_{i,EP\iota }^{total}={\tilde{DMI}}_{i}\;\times \;HdDays_{i,EP\iota }^{total} \end{array}$$

Following parameterization and simulation of animal variables, corresponding simulation of economic variables and calculations of relevant revenues and costs could proceed. Mean cattle prices ($/cwt; cwt = 45.4 kg = 100 lb) used as inputs were rounded to the nearest $0.25/cwt (USD). Only two market prices were simulated: corn price and fed cattle price, both following log-normal distributions (see Table 1 for means and SDs). Live-fed cattle prices were sourced from the “5 Area Weekly Weighted Average Direct Slaughter Cattle – Negotiated Purchases” report (LMIC; USDA AMS data) for steers sold live free-on-board on a negotiated cash basis. Fed cattle prices were allowed to vary for each EP, mimicking real-world conditions where producers face uncertain and fluctuating prices when marketing cattle on different weeks. We evaluated the correlation between live-fed cattle prices at four-time intervals separated by two weeks from the industry-reported data (corresponding to the four EPs), resulting in a 4 × 4 correlation matrix where nearer time points have stronger relationships. All EPs used the same mean live fed cattle price and parameterization (Table 1), so the distribution of prices across EPs was consistent across simulations, but prices varied within each iteration. Dressed-fed cattle base prices were calculated by dividing simulated live prices by 0.63, the mean ratio of live to dressed prices in the date range.

For each pen *i* and EPx (x ∈{1, 2, 3, 4}), revenue is computed for each of the three sale bases (*s* ∈ {live, dressed-cash, and grid}). Let ${\tilde{P}}_{i,EPx}^{live}$ be the simulated live-fed cattle price (*P*) per cwt, where prices for the *i*^*th*^ pen were correlated across EPx and were drawn from a log-normal distribution. Additionally, let ${\tilde{P}}_{i,EPx}^{dress}$ be the dressed fed cattle base price, where:


$$\begin{array}{l} {\tilde{P}}_{i,EPx}^{dress}=\;{\tilde{P}}_{i,EPx}^{live}÷0.63 \end{array}$$

Calculations of pen-level fed cattle revenue (**FCR**) for live and dressed-cash sale bases used animal counts, mean weights, and corresponding prices. These processes can be expressed as,


$$\begin{array}{l} FCR_{i,EPx}^{live}=Hd_{i,EPx}\times \;{\tilde{FBW}}_{i,EPx}\;\times \;\frac{{\tilde{P}}_{i,EPx}^{live}}{45.4\;kg} \end{array}$$

for live sale basis revenue and for dressed sale basis revenue:


$$\begin{array}{l} FCR_{i,EPx}^{dress}=Hd_{i,EPx}\;\times \;{\tilde{HCW}}_{i,EPx}\;\times \;\frac{{\tilde{P}}_{i,EPx}^{dress}}{45.4\;kg} \end{array}$$

For the grid sale basis, an adjustment to the base dressed price for premiums and discounts is required. Carcass premiums and discounts for QG, YG, and heavyweight carcasses (over 476 kg) were sourced from LMIC’s “5-Area Weekly Slaughter Cattle - Premiums and Discounts” report (USDA AMS data). Since premiums and discounts for YG and heavyweight carcasses are relatively constant, the YG grid and heavyweight discount were fixed across simulations at their approximate mean (Table 2). In contrast, QG grids fluctuate often. Instead of simulating QG grids, we selected three grids to evaluate low, middle, and high reward scenarios in terms of their preference towards higher QG (Grids 1 to 3 in Table 2). One of the three QG grids was randomly selected for use in each simulation iteration, with each grid used equally. The grids were based on the Choice-Select spread (i.e., Select discount, as USDA sets Choice to zero), selecting low, moderate, and high values at equal intervals. The simulation model combined all carcasses grading sub-Select into a single category, using the discount for Standard carcasses. Linear regressions (Proc REG, SAS 9.4) with Select discount as the independent variable and Prime premium or sub-Select (Standard) discount as the dependent variable were performed to estimate corresponding values for each QG grid. The resulting regressions used to determine Prime premiums and sub-Select discounts in QG Grids 1 to 3 were:

**Table 2.** Economic variables in the simulation model that were either fixed, regressed, or varied for sensitivity analysis that were incorporated for the evaluation of net return differences when feeding beef steers to later endpoints

Variable^1^	Value			Source
Dressed fed cattle base price,^2^ $/cwt	Regression			LMIC
Feed and yardage price,^3^ $/US ton	Regression			CattleFax, LMIC
Removal price,^4^ $/cwt HCW	Regression			Horton et al. (2021); LMIC
Treatment cost, $/n morbidity	23.60			USDA-NAHMS, 2013
Render fee, $/n mortality	40.50			Horton et al. (2024); Sparks Companies Inc. (2002)
Yield Grade grid, $/cwt				LMIC
1	5.00			
2	2.00			
3	0.00			
4	−10.00			
5	−14.00			
Heavyweight discount,^5^ $/cwt	−16.50			LMIC
Sensitivity analysis variables	Values			
Quality Grade grid, $/cwt	Grid 1	Grid 2	Grid 3	Source
Prime	15.75	16.50	22.75	Regression
Choice	0.00	0.00	0.00	LMIC
Select	−5.00	−17.50	−30.00	LMIC
Sub-Select	−19.50	−31.00	−41.00	Regression
	Rate 1	Rate 2	Rate 3	
Interest rate, fixed yearly %	5.0	7.0	9.0	Ag Credit Survey, Fed. Res. Bank of KS City

^1^Abbreviations: cwt = hundred-weight (45.4 kg or 100 lb); HCW = hot carcass weight.

^2^Simulated live fed cattle price divided by 0.63.

^3^Dry matter basis, U.S. ton = 907 kg or 2,000 lb; regression based on the simulated corn price.

^4^Regression for cull cow price based on the simulated live fed cattle price, then multiplied by 0.92.

^5^Carcasses weighing over 476 kg (1,050 lb).


$$\begin{array}{l} P_{\left(\pm SE\right)}^{Prime}=\;17.05_{\left(0.76\right)}+\;P_{\left(0.11\right)}^{Select}\times 0.35\;+\;P_{\left(0.004\right)}^{Select^{2}}\times 0.02\; \end{array}$$

where, $P^{Prime}\;\;\;=\;\;\;Prime\;\;\;premium,\;\;\;P^{Select}$ = Select discount, RMSE = 4.51, *R*^2^ = 0.13; and,


$$\begin{array}{l} P_{\left(\pm SE\right)}^{sub-Select}=\;-14.61_{\left(0.29\right)}+\;P_{\left(0.04\right)}^{Select}\times 1.00\;+\;P_{\left(0.001\right)}^{Select^{2}}\times 0.004 \end{array}$$

where $P^{sub-Select}$ = sub-Select discount, RMSE = 1.72, and *R*^2^ = 0.93. In both regressions, $P^{Select}$ and its quadratic term were significant (*P*-values < 0.05). As with fed cattle prices, premiums and discounts were on a $/cwt basis. The grid-adjusted price in each iteration is calculated by multiplying the premiums and discounts from the QG grid, YG grid, and heavyweight discount by the proportion of carcasses in each category and adding the result to the base price (Feuz et al., 2003). The grid adjustment can be defined as:


$$\begin{array}{l} GridAdj_{i,EPx}=\left({\tilde{QG}}_{i,EPx}\;⊙\;{\gamma QG\;}_{i}\right)+\left({\tilde{YG}}_{i,EPx}\;⊙\;\gamma YG\right)+\left(Heavy_{i,EPx}\;\times \;\gamma heavy\right) \end{array}$$

where ${\tilde{QG}}_{i,EPx}$ and ${\tilde{YG}}_{i,EPx}\;$ are the vectors of simulated carcass grades which are multiplied element-wise (denoted by ⊙) by the corresponding grid vectors, where ${\gamma QG\;}_{i}$ is one of the three randomly selected QG grids for pen *i*, and γYG is the fixed YG grid. The proportion of heavyweight carcasses ($Heavy_{i,EPx}$) is multiplied by the heavyweight discount (γheavy). Then, the pen-level grid revenue calculation can be expressed as:


$$\begin{array}{l} FCR_{i,EPx}^{grid}=Hd_{i,EPx}^{\;f\;inal}\;\times \;{\tilde{HCW}}_{i,EPx}\;\times \;\frac{\left({\tilde{P}}_{i,EPx}^{dress}+\;GridAdj_{i,EPx}\right)}{45.4\;kg} \end{array}$$

Simulated removed animals represented a small, but still relevant revenue source to account for, as it contributed to total revenue for EPι. Prices received for removals ($/cwt HCW) were estimated by multiplying cull cow prices (dressed Breaker cows over 227 kg [500 lb]) by 0.92 (Horton et al., 2021). Cull cow prices were estimated via a linear regression (Proc REG, SAS 9.4) with live-fed cattle price as the independent variable and cull cow price as the dependent variable, using data from LMIC’s “Weekly National Direct Cows and Bulls” report (USDA AMS data). The resulting regression was:


$$\begin{array}{l} P_{\left(\pm SE\right)}^{cow}=\;-27.18_{\left(4.42\right)}+\;P_{\left(0.03\right)}^{live}\times 1.30 \end{array}$$

where $P^{cow}\;\;\;=\;\;\;cull\;\;\;cow\;\;\;price,\;\;\;P^{live}$ = live fed cattle price (*P* < 0.01), RMSE = 17.21, and *R*^2^ = 0.75. The removal price was estimated using the fed cattle price from the prior EP as the predictor variable in the previous regression, simplifying the estimation process. The price received for removals ($P^{rem}$) in the *i*^*th*^ pen at EPι was:


$$\begin{array}{l} P_{i,EP\iota }^{rem}=\left(-27.18+\;{\tilde{P}}_{i,EP\left(x-1\right)}^{live}\times 1.30\right)\times 0.92 \end{array}$$

where there were no removals for EP1, which therefore has no removal price. We assumed removals weighed the simulated HCW of the prior EP for calculating revenue from these animals; therefore, revenue for removed animals ($R^{rem}$) for EPι could then be calculated as,


$$\begin{array}{l} R_{i,EP\iota }^{rem}=\;{\tilde{Rem}}_{i,EP\iota }\;\times \;{\tilde{HCW}}_{i,EP\left(x-1\right)}\times \frac{P_{i,EP\iota }^{rem}}{45.4\;kg} \end{array}$$

where removal revenue from prior EPι accumulates for subsequent EPι and total removal revenue is given as:


$$\begin{array}{l} R_{i,EP\iota }^{rem,total}=\;R_{i,EP\left(\iota -1\right)}^{rem,total}+\;R_{i,EP\iota }^{rem},\;with\;R_{i,EP1}^{rem}=0 \end{array}$$

Finally, total revenue for each sale basis ($TR_{i,EPx}^{s}$) for pen *i* at each EP was calculated as:


$$\begin{array}{l} TR_{i,EPx}^{s}=\;FCR_{i,EPx}^{s}+\;R_{i,EP\iota }^{rem,total},\;where\;TR_{i,EP1}^{s}=\;FCR_{i,EP1}^{s},\;as\;R_{i,EP1}^{rem}=0\; \end{array}$$

Costs were calculated for EPι only. In our model, opportunity cost refers to the cost associated with delaying revenue by marketing cattle at later EPs. One of three annual fixed interest rates (${Interest\;}_{i}$)—5%, 7%, and 9%—representing recent rates for operating loans (Ag Credit Survey, Federal Reserve Bank of Kansas City) were used to account for interest on feed and estimate opportunity costs. Each rate was randomly selected for each pen *i* (Table 2), with all rates used equally. Interest was not compounded. Opportunity cost was applied as a “penalty” to EPι for delaying marketing and not receiving revenue at EP1. To calculate it, the selected yearly interest rate was first multiplied by the additional DOF to reach each EPι (14, 28, or 42 d), divided by 365. The actual interest rate applied for each pen *i* was therefore:


$$\begin{array}{l} {Interest\;}_{i,EP\iota }={Interest\;}_{i}\;\times \;\frac{DOF_{EP\iota }}{365} \end{array}$$

Pen-level opportunity cost ($OC_{i,EP\iota }$) for the *i*^*th*^ pen was calculated based off EP1 FCR for each sale basis, where:


$$\begin{array}{l} OC_{i,EP\iota }=\;TR_{i,EP1}^{s}\times {Interest\;}_{i,EP\iota } \end{array}$$

Corn prices were sourced from the LMIC grain prices database (compiled from USDA AMS reports), specifically weekly prices for Kansas City #2 yellow corn. To approximate feed prices corresponding to simulated corn prices, we modeled a linear regression with feed and yardage price (**FYP**) as the dependent variable and corn price as the explanatory variable (Proc REG, SAS 9.4). Dry FYP was sourced from the CattleFax ration prices database (CattleFax, Centennial, CO) for the central plains region; since these prices are reported monthly, monthly corn prices were used for this regression. Feedlots use varying structures for feed markup and yardage—including feed markup with no yardage, no feed markup with yardage, and combinations thereof—so FYP is reported as a single sum. Yardage accounts for overhead costs associated with managing a feedlot (e.g., labor, equipment, utilities). The resulting regression was:


$$\begin{array}{l} FYP_{\left(\pm SE\right)}=\;26.80_{\left(36.18\right)}+\;P_{\left(14.87\right)}^{corn}\times 71.64\;-\;P_{\left(1.40\right)}^{corn^{2}}\times 2.97 \end{array}$$

where FYP = feed and yardage price, $P^{corn}$ = corn price, RMSE = 21.80, *R*^2^ = 0.88, and $P^{corn}$ (*P* < 0.01) and its quadratic term (*P* = 0.04) were significant. For pen *i*, ${\tilde{P}}_{i}^{corn}$ ($/bu; 1 bushel = 25.4 kg = 56 lb) was simulated from a log-normal distribution with the same value used across all EPι, which assumes grain was contracted for the pen beforehand (Table 1). The calculated FYP ($/dry US ton; 1 ton = 907 kg = 2,000 lb) for pen *i* was therefore:


$$\begin{array}{l} FYP_{i}=\;26.80+\;{\tilde{P}}_{i}^{corn}\times 71.64-{\left({\tilde{P}}_{i}^{corn}\right)}^{2}\times 2.97 \end{array}$$

Calculations of pen-level feed and yardage cost ($FYC_{i,EP\iota }$) for the *i*^*th*^ pen at each EPι incorporated FYP, DMI, and interest. For interest on feed, we used the same randomly selected interest rates (${Interest\;}_{i,EP\iota }$) for the *i^th^* pen at EPι as for opportunity cost, which was applied to 50% of the total pen feed and yardage cost to account for money borrowing. This final calculation for FYC can be expressed as:


$$\begin{array}{l} FYC_{i,EP\iota }=\left(\frac{FYP_{i}}{907\;kg}\;\times \;DMI_{i,EP\iota }^{total}\right)+\;\left(\frac{FYP_{i}}{907\;kg}\;\times \;DMI_{i,EP\iota }^{total}\;\times \;\frac{{Interest\;}_{i,EP\iota }}{2}\right) \end{array}$$

While removals provided a revenue source (albeit discounted compared to finished cattle), other health events—treatments and mortalities—resulted in costs (Table 2). Health costs ($HealthC_{i,EP\iota }$) for pen *i* at each EPι involved fixed treatment costs ($C^{treat}$) for any morbidity and rendering costs ($C^{rend}$) for any mortality. Treatment costs were fixed at $23.60 per treatment (USDA-NAHMS, 2013). For mortalities, besides the loss of revenue, a $40.50 per mortality fee for rendering was applied; this is an inflation-adjusted value (Sparks Companies Inc., 2002; Horton et al., 2024). Deriving $Morb_{i,EP\iota }^{total}$ and $Mort_{i,EP\iota }^{total}$ from $Health_{h,i,EP\iota }^{total}$, total pen-level health costs were calculated as:


$$\begin{array}{l} HealthC_{i,EP\iota }=\;C^{treat}\times Morb_{i,EP\iota }^{total}+\;C^{rend}\times Mort_{i,EP\iota }^{total} \end{array}$$

Lastly, total costs ($TC_{i,EP\iota }$) for pen *i* that were associated with extending DOF to EPι were therefore:


$$\begin{array}{l} TC_{i,EP\iota }=\;OC_{i,EP\iota }+\;FYC_{i,EP\iota }+\;HealthC_{i,EP\iota } \end{array}$$

Following all simulations and calculations of revenues and costs, final calculations could proceed. Pen-level net returns ($NR_{i,EP\iota }^{s}$) for pen *i* at EPι for each sale basis were calculated as:


$$\begin{array}{l} NR_{i,EP\iota }^{s}=\;TR_{i,EP\iota }^{s}-\;TC_{i,EP\iota } \end{array}$$

On its own, $NR_{i,EP\iota }^{s}$. has no real applicable value, as all costs prior to EP1 marketing are shared and therefore not included; it is simply total revenue minus costs associated with extended feeding. Meaning is derived once comparing back to EP1 revenue, yielding the computation of marginal net return differences ($\Delta NR_{i,EP\iota }^{\;pen,s}$), which denote the change in pen-level net return for pen *i* that occurred while alternatively feeding to EPι for each sale basis:


$$\begin{array}{l} \Delta NR_{i,EP\iota }^{\;pen,s}=\;NR_{i,EP\iota }^{s}-\;TR_{i,EP1}^{s} \end{array}$$

For interpretation, these values were converted to be expressed as “per animal sold,” which includes both removals and finished steers. This final calculation was:


$$\begin{array}{l} \Delta NR_{i,EP\iota }^{hd,s}=\;\frac{\Delta NR_{i,EP\iota }^{\;pen,s}}{Hd_{i,EP\iota }+\;Rem_{i,EP\iota }^{total}} \end{array}$$

where $Rem_{i,EP\iota }^{total}$ was derived from the previously defined, $Health_{h,i,EP\iota }^{total}$.

### Simulation model implementation

The simulations were performed using Python (Python Software Foundation, version 3.12.3), executed within a JupyterLab (Jupyter Team, version 4.2.2) environment. The primary Python packages utilized for the stochastic simulations were “NumPy” (version 2.0.0) and “SciPy” (version 1.14.0) for numerical computations, statistical distributions, and correlation matrices. When necessary, a Cholesky decomposition was used to generate correlated random variables, applying the decomposition to correlation matrices tailored to each variable’s distribution (e.g., normal, logistic) to maintain realistic correlations among simulated outcomes. Additionally, “Pandas” (version 2.2.2) was used for data manipulation, and “Matplotlib” (version 3.9.0) along with “Seaborn” (version 0.13.2) was employed for data visualization. To determine the total number of simulations, model convergence was defined by stabilization at specified percentiles. This was evaluated by initially performing 135,000 simulations and increasing the number by 45,000 until the values at percentiles (0.5, 2.5, 10, 25, 75, 90, 97.5, and 99.5) remained within a 1% change from the previous simulation count. Convergence on the median was not required because as certain values approach zero, small-dollar changes can result in percentage changes exceeding 1%, which could prevent convergence or necessitate an impractically large number of simulations.

### Data evaluation and visualization

Analyses and reporting of results were mainly descriptive, as minimal statistical tests are appropriate for simulated data. For our primary objective, distributions of net return differences for EP2, EP3, and EP4 were visualized with overlaid density plots, along with boxplots with rainclouds (strip plots). Due to the large number of simulations, rainclouds used a random subsample of the data for improved visualization, while boxplots used the full dataset. Specific percentile values are also reported in tabular formats for precision.

To address our secondary objective of identifying variables that most influenced net return differences, we used conditional random forest models to compute variable importance scores. Conditional random forests, which implement a conditional permutation scheme for computation of importance scores, mitigate biases from correlated predictor variables better than traditional random forests (Strobl et al., 2008). While random forests are primarily employed for predictive purposes, they can be a tool for causal inference through importance scores with certain assumptions (van der Laan, 2006). Models were built using the “party” package (version 1.3-15; Strobl et al., 2008) and used “permimp” (version 1.0-2; Debeer and Strobl, 2020) for faster computation importance scores, in R (version 4.4.1; R Core Team, 2024), which was called within a JupyterLab environment using the “rpy2” (version 3.5.16) interface for the execution of R code embedded in Python processes. We constructed nine models—one for each EP within each of the three sale bases—and summarized the variable importance scores within each sale basis. Only importance scores were evaluated; predictive accuracy was not assessed, as our purpose was to visualize and rank the variables by importance for their impact on net returns. Due to processing demands, a random subsample of the simulated data was used, with sample size and number of trees increased until variable importance rankings stabilized. Models used a fixed number of randomly sampled variables selected as candidates at each split (node) in the trees (“mtry” *n* = 4), which was the square-root of the number of parameters included, rounded up. Predictor variables for live and dressed-cash sale bases included DMI, final weight, weight gain, number of treatments, removals, mortalities, corn price, fed cattle price, price difference compared to EP1, interest rate, and opportunity cost. For the grid sale basis, additional variables were percent heavyweight carcasses, percent Choice or better carcasses (proxy for QG), percent YG 4 and 5 carcasses (proxy for YG), and the QG grid. After model execution, raw scores were scaled from 0 to 100, and variables were ranked by importance.

Lastly, we categorized the simulated data based on the magnitude and direction of net return differences for EP2, EP3, and EP4 to visualize changes in variables of interest and identify conditions under which feeding to a later EP was advantageous or not. Net return differences were grouped into five categories: less than $−25/animal, $−25 to $−5/animal, $−5 to $5/animal, $5 to $25/animal, and greater than $25/animal compared to EP1. This framework allowed us to evaluate the frequency of simulations in each category and to descriptively assess variable values when net return differences ranged from negative to positive.

## Results

Model convergence was achieved with 360,000 simulations. Distributions of the simulated economic, cattle performance, carcass characteristics, and health outcomes are reported in Supplementary Tables 1 to 4.

**Figure 1.**
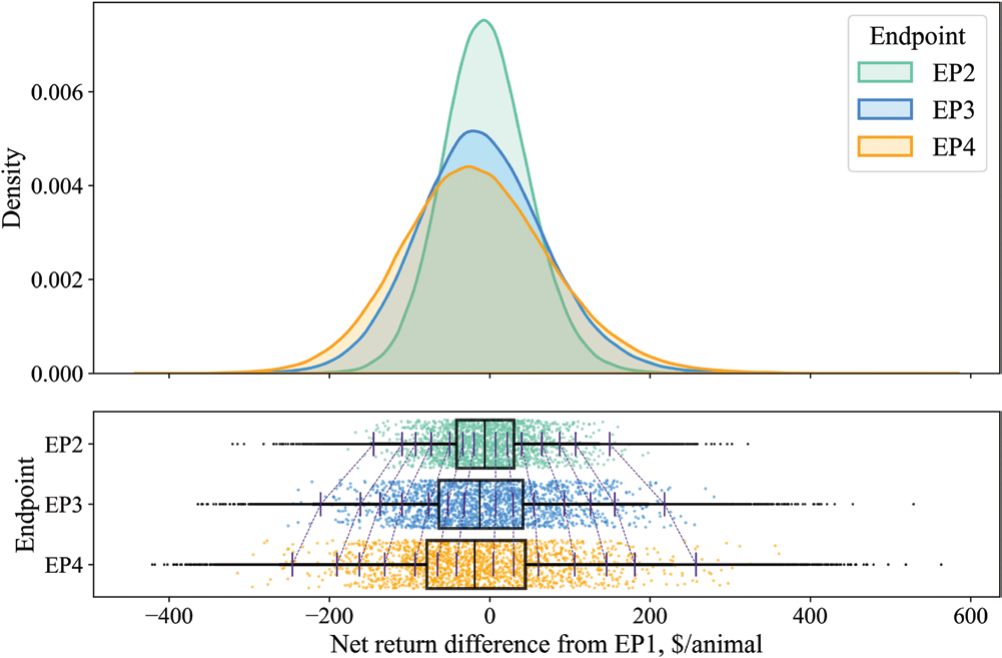
Distributions of net return differences comparing later-fed endpoints (EPs) to EP1 from a stochastic simulation model of beef feedlot steers marketed on a live basis (*n* = 360,000 simulations). Bars with connecting dotted lines are percentiles (from left to right: 0.5, 2.5, 5, 10, 20, 30, 40, 60, 70, 80, 90, 95, 97.5, and 99.5th percentiles, respectively). Raincloud plots show a random subsample of the data for visualization purposes, while boxplots and overlaid density plots use the full dataset.

**Figure 2.**
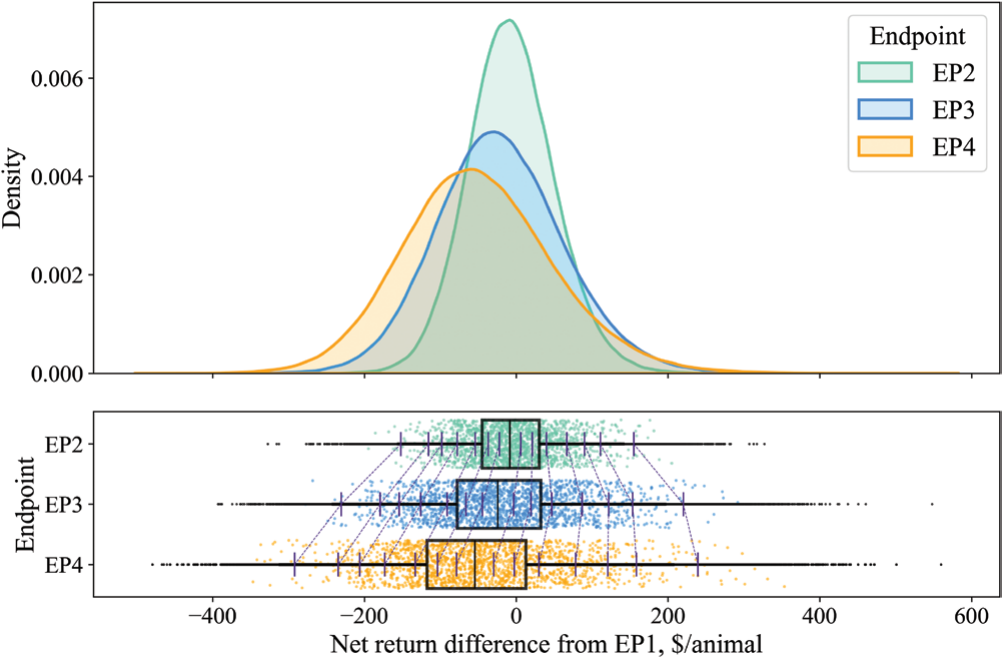
Distributions of net return differences comparing later-fed endpoints (EPs) to EP1 from a stochastic simulation model of beef feedlot steers marketed dressed with a premium and discount-based grid (*n* = 360,000 simulations). Bars with connecting dotted lines are percentiles (from left to right: 0.5, 2.5, 5, 10, 20, 30, 40, 60, 70, 80, 90, 95, 97.5, and 99.5th percentiles, respectively). Raincloud plots show a random subsample of the data for visualization purposes, while boxplots and overlaid density plots use the full dataset.

Figures 1 and 2 illustrate the distributions of net return differences for EP2, EP3, and EP4 compared to EP1 when marketing on live and grid sale bases, respectively. Median net return differences were negative for EP2, EP3, and EP4, under both sale bases, indicating that feeding to later EPs was more frequently disadvantageous than not. When live marketing, net return differences were positive (greater than $0/animal) in 45.4%, 43.7%, and 42.0% of the simulations for EP2, EP3, and EP4, respectively. When grid marketing, net return differences were positive in 44.1%, 38.4%, and 29.1% of the simulations for EP2, EP3, and EP4, respectively. As expected, EP2 exhibited a narrower distribution around the median, while EP3 and EP4 showed wider distributions, suggesting increased potential gains but also greater risk of substantial losses with extended feeding. In contrast to live marketing (Figure 1), EP3 and EP4 had more pronounced shifts toward negative net returns when marketing on a grid basis (Figure 2). Table 3 presents the percentile values of these distributions for both sale bases. When marketing on a live basis, net return differences for EP2, EP3, and EP4 became positive by the 60th percentile. On a grid basis, net returns turned positive by the 60th, 70th, and 80th percentiles for EP2, EP3, and EP4, respectively. When net returns were negative at any given percentile, returns declined consistently with increasing EP for both sale bases. When live marketing, EP3 net returns exceeded EP2 above the 60th percentile, and EP4 surpassed both EP2 and EP3 above the 70th percentile. In contrast, on a grid basis, EP3 did not surpass EP2 until the 80th percentile, and EP4 only exceeded EP2 and EP3 at the 97.5th percentile. Lastly, EP2 distributions were similar between live and grid marketing, with slightly lower negative values on a grid basis that narrowed and ultimately resulted in slightly higher positive values at greater percentiles. Conversely, EP3 and EP4 net returns were almost always lower at every percentile on a grid basis compared to live. Supplementary Figure 1 and Supplementary Table 5 show net return distributions when marketing on a dressed-cash basis. Contrary to live and grid models, median net return differences compared to EP1 were positive for each later-fed EP and increased with each incremental EP.

**Table 3.** Tabular percentile values of the distributions of net return differences comparing later-fed endpoints (EPs) to EP1 from stochastic simulation models of beef feedlot steers^1^

	Percentile														
Net return difference from EP1, $/animal	0.5	2.5	5	10	20	30	40	50	60	70	80	90	95	97.5	99.5
Live sale basis															
EP2	−144.95	−109.44	−92.43	−72.99	−49.94	−33.63	−19.51	−6.18	7.28	21.96	39.76	65.24	87.28	107.19	149.70
EP3	−210.92	−161.34	−136.93	−109.24	−76.16	−52.23	−31.84	−12.45	7.42	29.13	55.14	93.02	125.76	156.02	218.06
EP4	−246.36	−190.51	−162.69	−130.99	−93.11	−65.31	−41.47	−18.72	4.53	30.05	60.82	105.94	145.64	181.05	257.42
Grid sale basis															
EP2	−152.03	−115.44	−97.99	−77.79	−53.87	−36.85	−22.18	−8.16	5.95	21.33	40.02	66.78	89.89	110.94	154.98
EP3	−230.65	−179.29	−154.30	−125.79	−91.17	−66.06	−44.58	−24.18	−3.38	19.61	46.95	86.98	121.78	153.37	220.16
EP4	−291.90	−234.40	−206.06	−173.12	−133.04	−103.80	−78.51	−54.35	−29.62	−2.58	30.11	78.24	120.54	158.46	239.29

^1^Stochastic simulation models (*n* = 360,000 simulations) parameterized animal performance, carcass characteristics, health, as well as economic variables to evaluate differences in net returns when feeding steers to later endpoints under variable conditions.

Table 4 shows categorized range frequencies of net return changes for EP2, EP3, and EP4 compared to EP1 for live and grid sales. On a live basis, net returns exceeding $5/animal compared to EP1 occurred in 41.6%, 41.2%, and 39.8% of the simulations for EP2, EP3, and EP4, respectively. Conversely, a loss of $−5/animal or more occurred in 50.9%, 53.8%, and 55.9% of the simulations for EP2, EP3, and EP4, respectively. When marketing on a grid basis, more than $5/animal was returned for later-fed EPs compared to EP1 in 40.7%, 36.2%, and 27.5% of the simulations for EP2, EP3, and EP4, respectively. In contrast, a loss of -$5/animal or more occurred in 52.3%, 59.3%, and 69.2% of the simulations for EP2, EP3, and EP4, respectively, when marketing on a grid.

**Table 4.** Frequency statistics of categorized net return differences compared to endpoint 1 (EP1) for feedlot steers fed to later EP in a stochastic simulation model when marketed live or dressed with a premium and discount-based grid^1^

	Live basis			Grid basis		
Net return difference category, %	EP2	EP3	EP4	EP2	EP3	EP4
<$−25/animal	36.0	43.5	47.2	38.0	49.6	61.8
$−25 to $−5/animal	14.9	10.3	8.7	14.3	9.7	7.4
$−5 to $5/animal	7.4	5.0	4.3	7.1	4.6	3.4
$5 to $25/animal	13.5	9.4	8.0	12.8	8.4	6.1
>$25/animal	28.1	31.8	31.8	27.9	27.8	21.4

^1^Stochastic simulation models (*n* = 360,000 simulations) parameterized animal performance, carcass characteristics, health, as well as economic variables to evaluate differences in net returns when feeding steers to later endpoints under variable conditions.

**Figure 3.**
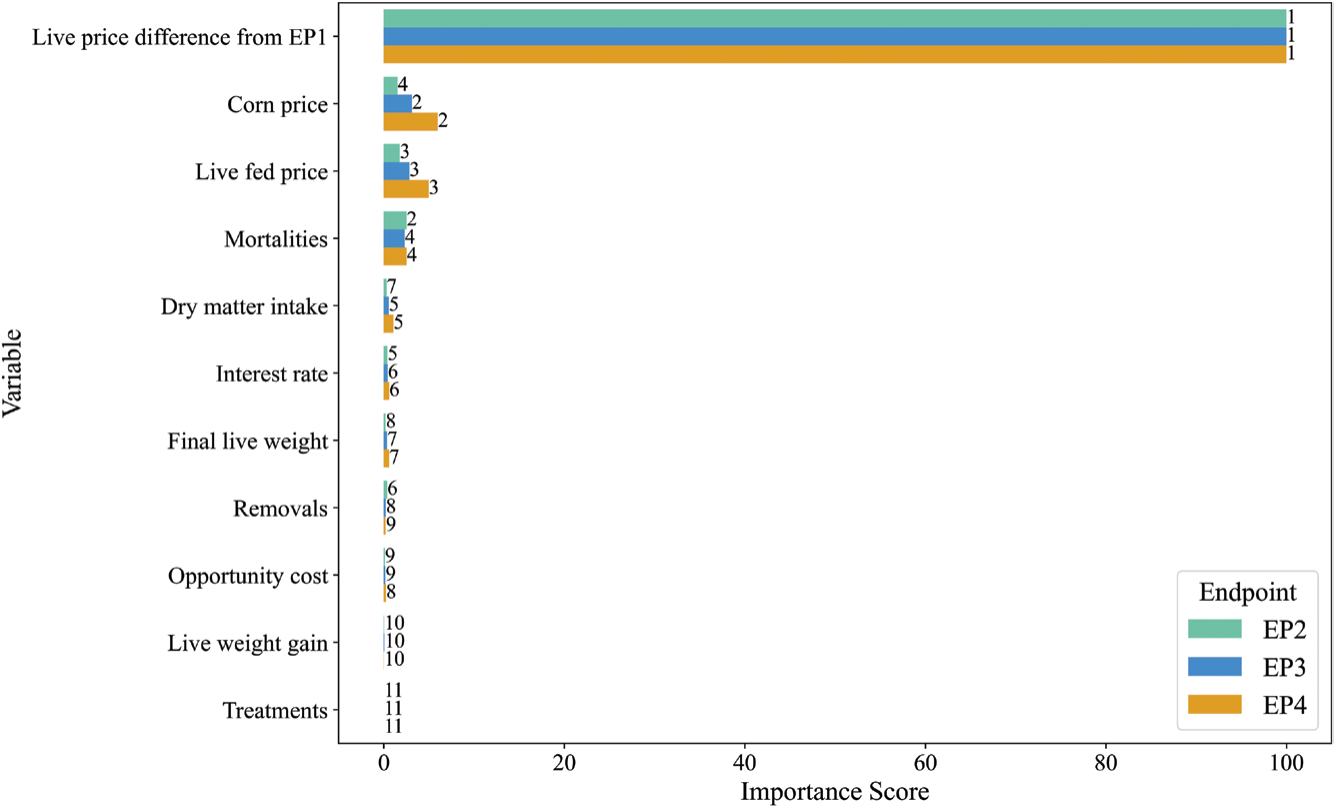
Conditional variable importance scores from conditional random forest models to estimate the relative importance of the specified variables influencing net return differences of later-fed endpoints (EP2, EP3, and EP4) compared to EP1 when marketing feedlot steers on a live sale basis in the stochastic simulation model. Data labels indicate the ranking from most to least important (1 to 11, respectively) within each EP.

For ranking conditional variable importance scores, the conditional random forest models were deemed stable using a 12.5% random subsample of the simulated data (*n* = 45,000 samples) and 200 trees. The conditional importance scores are determined via permutation accuracy, which in essence evaluates changes in predictive accuracy of the model with and without each variable included (Strobl et al., 2008). Raw scores do not necessarily have a straightforward or intuitive meaning (hence scaling to 100); therefore, only relative magnitudes and rankings of the scores are compared. For visualization, variables are sorted by their EP3 score ranking. When marketing live (Figure 3), the most important variable, by a substantial margin, was the live-fed cattle price difference—the marginal difference in fed cattle price received for each EP compared to EP1. The next “cluster” of variables with higher importance included corn price, the live-fed cattle price itself, and the number of mortalities. In terms of rankings (from high to low, 1 to 11; Figure 3), corn price and live-fed cattle price were more important for EP3 and EP4 than the number of mortalities. Conversely, mortalities were potentially more influential for EP2 net return differences than corn price or live-fed cattle price. Variables such as DMI, interest rate, and final live BW had marginal to low importance scores, while removals, opportunity cost, live weight gain rate, and the number of treatments were of minimal importance.

**Figure 4.**
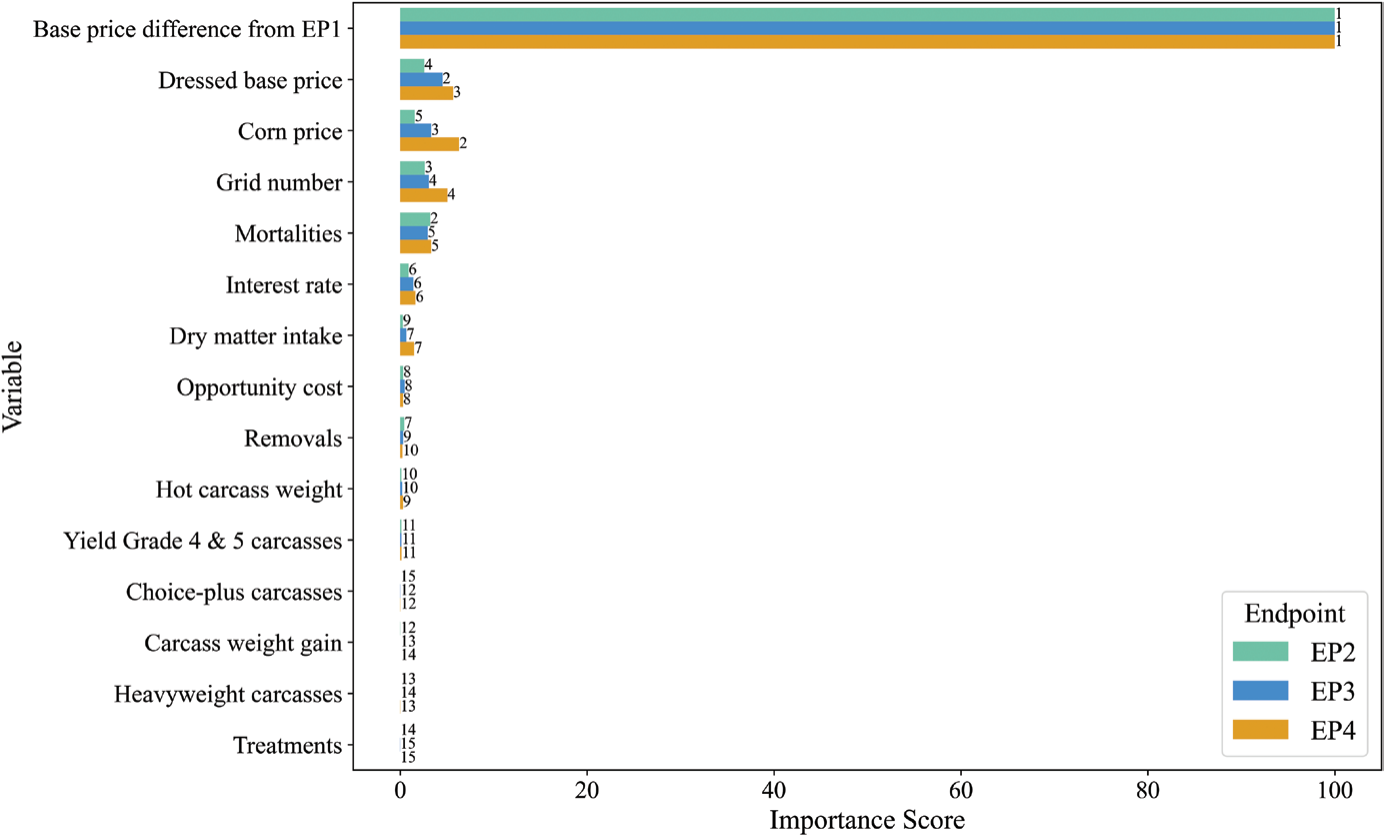
Conditional variable importance scores from conditional random forest models to estimate the relative importance of the specified variables influencing net return differences of later-fed endpoints (EP2, EP3, and EP4) compared to EP1 when marketing feedlot steers on a dressed basis with a premium and discount-based grid in the stochastic simulation model. Data labels indicate the ranking from most to least important (1 to 15, respectively) within each EP.

When marketing on a grid basis (Figure 4), variable importance scores were similar to live marketing, with a few additions. The difference in dressed-fed cattle price compared to EP1 remained the most critical variable. The next tier encompassed the corn price, dressed-fed cattle base price, and the number of mortalities, and now included the QG grid number. Rankings (from high to low, 1 to 15; Figure 4) in this cluster were consistent for EP3 and EP4, with minor switches between corn price and dressed base price. Mortalities were again of greater relative importance for EP2 than other variables in this cluster, contrasting EP3 and EP4. Interest rate and DMI were arguably the only two variables with marginal importance when grid marketing, depending on the EP. The remaining variables, including the percentage of carcasses grading Choice or better (Choice-plus), the percentage of YG 4 and 5 carcasses, and the percentage of heavyweight carcasses, were of minimal importance. Variable importance scores when marketing on a dressed-cash basis are shown in Supplementary Figure 2.

**Figure 5.**
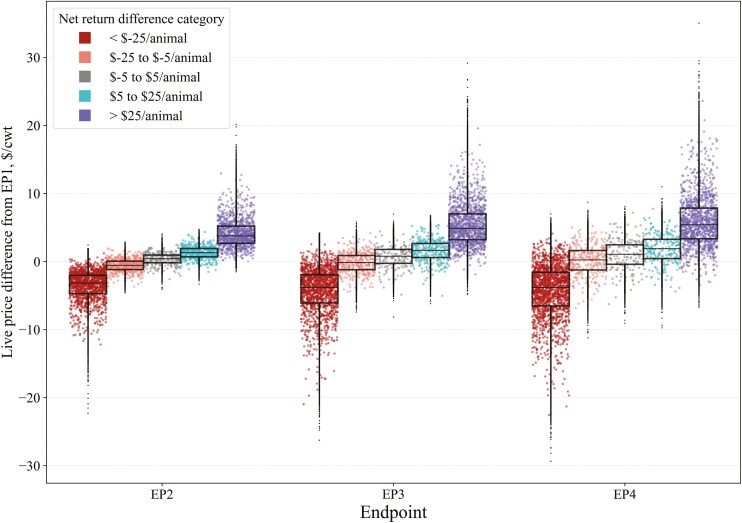
Distribution of simulated live-fed cattle price differences compared to endpoint 1 (EP1), when categorized by net return differences (compared to EP1) when feeding steers to later EP and marketed live in a stochastic simulation model. Raincloud plots show a random subsample of the data for visualization purposes, while boxplots use the full dataset.

Since the difference in fed cattle prices compared to EP1 was the most influential factor on net return differences, these price differences are visualized in Figure 5, which shows the distributions of live fed cattle price changes from EP1 across each EP when marketing on a live basis; additional descriptive statistics are in Supplementary Table 6. As expected, receiving lower fed cattle prices than EP1 at later EPs was associated with lower net returns and vice versa. As EPs increased, the price change distributions within net return categories (except for the <$−25/animal category) shifted upward, indicating that larger price increases were more often needed for later-fed EPs to achieve higher net returns compared to EP1. Additionally, the spread of live-fed cattle price differences widened with increasing EPs, reflecting increased price volatility as feeding horizons lengthen.

**Figure 6.**
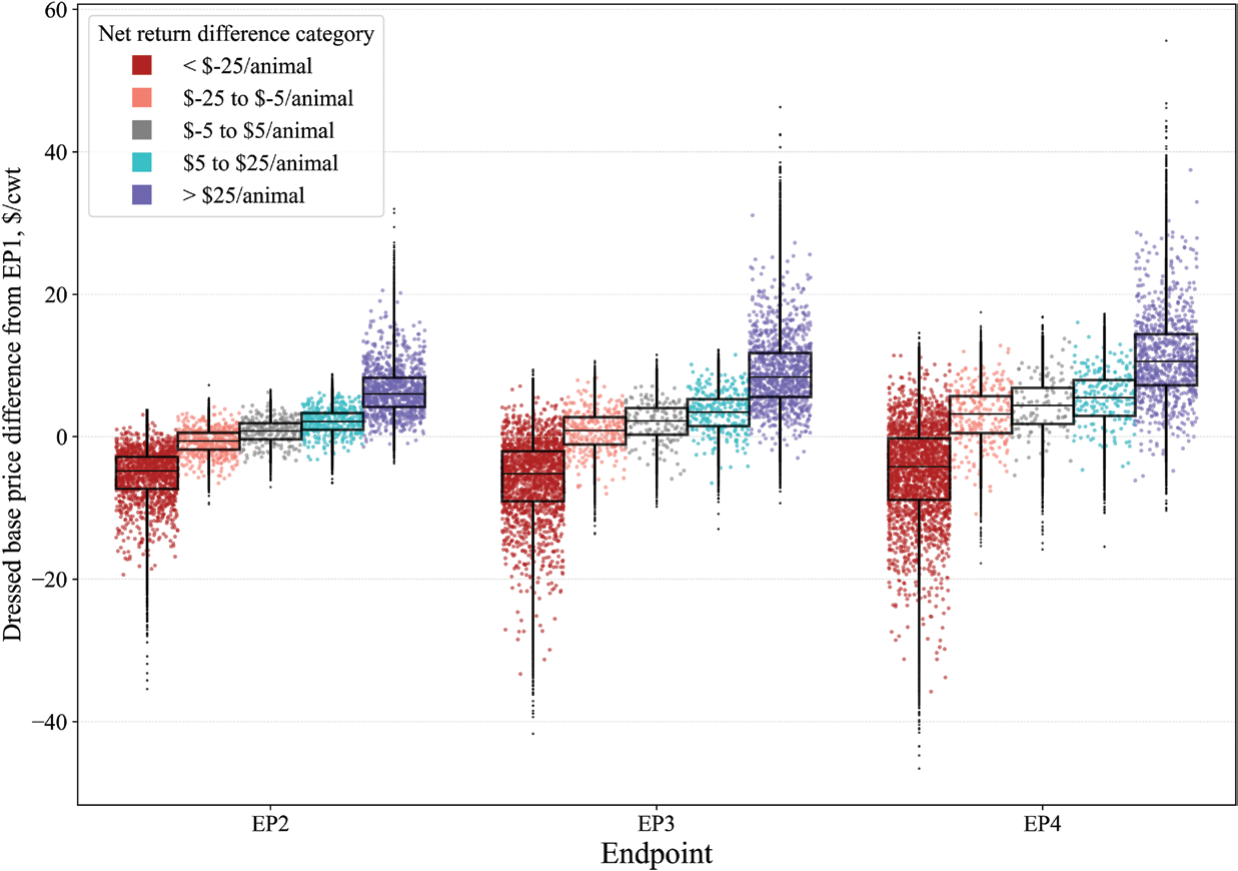
Distribution of simulated dressed feed cattle base price differences compared to endpoint 1 (EP1), when categorized by net return differences (compared to EP1) when feeding steers to later EP and marketed dressed with a premium and discount-based grid in a stochastic simulation model. Raincloud plots show a random subsample of the data for visualization purposes, while boxplots use the full dataset.

Grid marketing results (Figure 6) are similar to live marketing. Except in the <$−25/animal category, the distributions of dressed-fed cattle base price changes within net return categories also shifted upward and widened as EPs increased. A notable difference in grid marketing is that larger differences in the dressed base price were more often required for net returns to increase with later EPs compared to live marketing. For example, the median live-fed cattle price differences (vs. EP1) for the $5 to $25/animal net return category were $1.31, $1.65, and $1.89/cwt for EP2, EP3, and EP4, respectively. In contrast, when marketing on a grid, the median dressed base price differences were $2.15, $3.46, and $5.51/cwt for EP2, EP3, and EP4, respectively (Figure 6; Supplementary Table 6). These values not only incrementally increased more with each subsequent EP when marketing on a grid but were also substantially higher overall than live marketing. While dressed-fed cattle prices are inherently higher than live prices due to the live-to-dressed conversion factor (0.63), the median values specified surpass this ratio. The most extreme examples occurred with EP4, where much higher price changes were frequently required to observe increased net returns. For instance, even in the net return category where $−25 to $−5/animal was lost, the median dressed base price difference was $3.19/cwt, with an interquartile range (**IQR**) of $0.51 to $5.70/cwt (i.e., all higher prices than EP1). While some instances of near-zero or negative price changes from EP1 resulted in increased net returns for later-fed EPs, these occurrences were infrequent. Distributions of dressed base price differences when marketing on a dressed-cash basis are in Supplementary Figure 3.

Although the difference in fed cattle prices received was the most influential factor on net returns, other important variables also showed notable characteristics within net return categories. Supplementary Tables 6 and 7 provide descriptive statistics for all common variables used in the random forest models (Figures 3 and 4), categorized by net return category and EP. The most noteworthy observation is that the descriptive statistics align with the implications of the variable importance scores. For instance, as net return categories moved toward more positive returns, fed cattle prices increased, while corn prices decreased. The median and IQR values for the number of mortalities remained relatively unchanged across net return categories; however, the mean number of mortalities tended to decrease as net returns improved. Variables previously described as marginally important, such as DMI and interest, showed slight differences across net return categories. In contrast, variables identified as the least important (e.g., final live BW or HCW, weight gain, treatments) exhibited very similar statistics across net return categories, supporting their lower importance.

Additional variables included in the conditional random forest models for the grid sale basis only were grid number, percent of YG 4 and 5 carcasses, percent of Choice and Prime carcasses, and percent of heavyweight carcasses. The grid number, in particular, was part of the cluster of variables with higher importance (after the dressed base price difference). Table 5 shows frequency statistics for each grid number within net return difference categories and EP. For EP2, EP3, and EP4, grid number 2 occurred relatively consistently, about 33% of the time, across all net return categories. However, as net return increased, the frequency of grid number 1 decreased, while the frequency of grid number 3 increased, regardless of EP (Table 5). While a higher grid number was not necessarily required to achieve higher net return categories (grid number 1 occurred in the >$25/animal category 28.9%, 28.5%, and 27.3% of the time for EP2, EP3, and EP4, respectively), grid number 3, which gives the greatest reward to higher QGs, was more commonly associated with higher net return differences. Specifically, grid number 3 occurred 38.1%, 38.4%, and 39.9% of the time for EP2, EP3, and EP4, respectively, in the >$25/animal category. While grid number was important for QG premiums and discounts, it was interesting to note that the percentage of Choice-plus carcasses, YG 4 and 5 carcasses, and heavyweight carcasses had minimal importance in the conditional random forest models (Figure 4). Descriptive statistics for these variables are available in Supplementary Table 8. Overall, the descriptive statistics align with the variable importance scores, as there were minimal differences—unlikely to be biologically meaningful—when evaluating these carcass characteristics across net return categories and within EPs.

**Table 5.** Frequency statistics of grid numbers and their occurrence within net return difference categories for later-fed endpoints from a stochastic simulation model of feedlot steers marketed on a premium and discount-based grid^1^

	Grid number,^2^ %		
Net return difference category	1	2	3
Endpoint 2			
<$−25/animal	37.3	33.5	29.2
$−25 to $−5/animal	33.5	33.6	32.9
$−5 to $5/animal	33.1	33.2	33.7
$5 to $25/animal	31.2	33.4	35.4
>$25/animal	28.9	33.0	38.1
Endpoint 3			
<$−25/animal	36.8	33.5	29.7
$−25 to $−5/animal	32.4	33.5	34.1
$−5 to $5/animal	31.6	33.3	35.1
$5 to $25/animal	30.9	33.1	36.0
>$25/animal	28.5	33.1	38.4
Endpoint 4			
<$−25/animal	36.3	33.5	30.2
$−25 to $−5/animal	31.3	33.1	35.6
$−5 to $5/animal	29.4	33.3	37.4
$5 to $25/animal	29.5	33.5	37.0
>$25/animal	27.3	32.8	39.9

^1^Stochastic simulation models (*n* = 360,000 simulations) parameterized animal performance, carcass characteristics, health, as well as economic variables to evaluate differences in net returns when feeding steers to later endpoints under variable conditions.

^2^Each grid number awarded different Quality Grade premiums and discounts, with each based on the Choice-Select spread; grid number 1 had the smallest Choice-Select spread, while grid number 3 had the largest Choice-Select spread.

Given the limited literature on the opportunity cost of extending DOF in feedlot steers, we provide a more detailed description of our findings. Descriptive statistics of opportunity cost for live and grid sale bases, broken down by net return category within each EP, are provided in Supplementary Table 6. However, these values are not distinguished by interest rate. When live marketing, for each additional 14 DOF beyond EP1, the median opportunity cost (95% inter-percentile range [**95IPR**; 2.5th to 97.5th] in parentheses) was $4.23 ($3.06 to $5.85), $5.92 ($4.28 to $8.18), and $7.60 ($5.50 to $10.53) per animal at interest rates of 5%, 7%, and 9%, respectively. When grid marketing, the median opportunity cost for each additional 14 DOF beyond EP1 was $4.20 ($2.99 to $5.86), $5.87 ($4.18 to $8.20), and $7.55 ($5.38 to $10.56) per animal at interest rates of 5%, 7%, and 9%, respectively.

Because no direct comparisons were made between marketing scenarios, nor between EP2, EP3, and EP4 (all comparisons were with EP1 within each sale basis), between-variable correlations (e.g., live BW gain and HCW gain) were not impactful and altering the matrices did not affect net return distributions. However, within-variable correlations (i.e., YG, QG, and fed cattle prices across EPs) were critical and had strong impacts on the results. Therefore—for reproducibility purposes—these matrices are provided in Supplementary Table 9.

## Discussion

Results presented here are applicable to contemporary feedlot steer populations under recent market conditions. Numerous research efforts have evaluated the performance and carcass changes of feedlot steers and heifers when extending DOF. Much of this work has been pooled and summarized in a comprehensive multi-trial analysis (Galyean et al., 2023). However, the studies included in Galyean et al.’s (2023) analysis may not necessarily reflect current standards for cattle populations that have been progressively fed to heavier EPs (approximately 15% HCW increase since 2004; LMIC) with higher QG and YGs (approximately 49% increase [28 percentage points] of carcasses grading Choice-plus since 2004; LMIC). In contrast, the trial data used to support parameterization of the simulation model represents contemporary cattle populations (EP1) and evaluates feeding to more extreme EPs than previously reported. A key consideration is that our findings do not imply that cattle feeders have been overfeeding or that they should market steers earlier, as earlier than the standard marketing endpoint was not evaluated. Instead, our results suggest that feeding beyond recent standards may not frequently be advantageous at the industry level. Similarly, because our model is based on industry-reported data, it carries an industry-level interpretation and should not be taken to reflect individual-level risk. Producers with different baseline cattle populations, EPs, or grid pricing and marketing structures may experience risks that deviate from the industry average. Although one might argue for using a wider date range for cattle and grain market prices to illustrate longer-run distributions of potential outcomes, this introduces challenges like apparent structural changes in premiums and discounts for grid-marketed cattle, particularly for heavyweight and YG 4 and 5 discounts, which have substantially decreased in recent years (LMIC). Therefore, we focused on a shorter time frame reflecting contemporary populations and economic conditions, recognizing that models like ours may require adjustments for future market shifts.

Feeding to later EPs resulted in wider distributions of net returns across all marketing schemes, indicating increased risk and uncertainty when extending DOF. Much of this variability was driven by changes in fed cattle prices compared to EP1, reflecting market volatility when marketing on different dates. Varying prices and other mutable simulated variables naturally lead to increased variability, especially as EPs are extended. The distributions showed that the risk of reduced returns was lower when marketing on a live basis compared to a grid basis. However, it is important to note that “net return” in this model does not equate to profit. While grid marketing generally offers greater potential for revenue and profit than live marketing, it is more variable and highly dependent on cattle quality (Schroeder and Graff, 2000). Previous studies suggest that when extending DOF, marketing on a carcass basis is more advantageous than live (Wilken et al., 2015; Horton et al., 2024). Our model does not determine whether profit increased or decreased between live and grid marketing; it quantifies net return differences within each sale basis. Although grid marketing more frequently resulted in reduced net returns compared to live marketing, it may still have been more profitable. Since the model does not distinguish differences in EP1 baseline profits between marketing schemes, it does not infer which sale basis offered higher returns but only contrasts net return differences within each sale basis. Nevertheless, a distinct pattern emerged: net return reductions were more frequent and substantial with each successive EP when marketing on a grid basis.

While the weights of steers at EP1 reflected industry averages (also mirroring the trial data), it was necessary to use estimates from the trial data to specify QG and YG distributions and their shifts for later-fed EPs. Steers in the trial data reflected Texas QG averages (LMIC), which tend to be lower (fewer Choice and Prime carcasses) than other regions and the national average. Industry averages for QGs are not distinguished by sex and may be biased due to combined reporting with heifers, which tend to have higher marbling scores and QGs than steers (Choat et al., 2006). Although the median simulated QGs aligned most closely with Texas-fed cattle, wide distributions of grades were simulated, including values reflective of higher-grading regions in the United States, albeit less frequently. Therefore, both high- and low-grading conditions were accounted for in the simulation model. Even with grid pricing and dressed marketing, HCW (Johnson and Ward, 2005; Tatum et al., 2006, 2012) and base-fed cattle prices (McDonald and Schroeder, 2003; Harri et al., 2009) remain the most influential factors on revenue, with QG and YG importance increasing as the Choice-Select spread widens (Johnson and Ward, 2005; Tatum et al., 2006).

Overall, when grid marketing and feeding to later EPs, the shift toward higher QGs did not offset the corresponding higher YGs and increased heavyweight carcasses; the premiums did not outweigh the discounts. In contrast, cattle marketed on a dressed-cash basis—reported only in the Supplementary Material due to its limited use in the United States—showed that all EPs had positive net return differences compared to EP1 at the median, with these differences increasing as EPs were extended. The only difference between dressed-cash and grid sale bases was the inclusion of carcass-based premiums and discounts in the grid sale basis. This provides evidence that grid price adjustments on average negatively impacted revenue when feeding to later EPs, explaining the more frequent need for higher dressed base prices from EP1 to achieve greater net returns. This observation differs from Horton et al. (2024), who found that grid revenue (from grid adjustments, excluding base price) increased for feedlot heifers fed for 21 or 42 d beyond a baseline EP, implying that QG premiums outweighed discounts for YG and heavyweight carcasses. However, their study focused on heifers, which had minimal heavyweight carcasses and were fed to lighter EPs, further from current industry standards.

Despite this, QG (Choice-plus), YG (4 and 5), heavyweight carcasses, and even HCW were of low relative importance in our models. This does not suggest these variables are not critical revenue determinants; that is not the question our simulation model addresses. Rather, it indicates that when extending DOF beyond EP1, neither HCW nor any of the simulated carcass characteristics appreciably influenced the marginal net return changes across EPs. These variables might be more important in different contexts (e.g., simulations with a high baseline HCW for EP1 should result in more total revenue than those with a lower baseline HCW). However, since our primary research question focused on comparisons within individual simulations back to EP1, there was no evidence that these variables strongly impacted the net return difference. In other words, when making pen-level decisions, it appears more prudent for producers to prioritize factors other than weight gain or carcass grade changes when deciding whether to extend the feeding period for a pen of steers beyond their anticipated EP.

In the stochastic simulation model, economic factors accounted for three of the four most important variables when live marketing and four of the top five when grid marketing. This suggests that economic conditions are the primary drivers for deciding whether to extend the DOF of feedlot steers beyond a standard EP, rather than the characteristics of the steers themselves. Seasonal trends in United States cattle and grain prices are well-documented (Mark et al., 2002; Peel and Meyer, 2002; Sørensen, 2002; Ji et al., 2017). Although seasonality was not explicitly included in our model, one can infer that extending DOF may not be advantageous when fed cattle prices are expected to drop, given the high importance of fed cattle price changes in our model. For example, the most extreme price declines typically occur between May and July, before prices begin to rise again in August or September (Mark et al., 2002; Peel and Meyer, 2002). Corn prices also tend to peak in early summer (LMIC; Sørensen, 2002), compounding the disincentive to add DOF during this period. Conversely, conditions may favor extending DOF when fed cattle prices are expected to rise, as is historically typical of fall through early spring (LMIC; Mark et al., 2002; Peel and Meyer, 2002). Futures markets could serve as indicators to help producers anticipate higher fed cattle prices and consider delaying marketing. Regardless of the strategy, price volatility should be expected, and risk management strategies are advisable (Harri et al., 2009).

For both live and grid marketing, after the fed cattle price difference, the most important economic variables were, in no particular order, the fed cattle price (live or dressed base), corn price, and for grid sales, the QG grid. Previous research indicates that fed cattle price is the most critical factor for profit determination in the feedlot, followed by feeder cattle prices and corn prices (Mark et al., 2000). As previously mentioned, the importance of the dressed base price and QG grid prices for grid marketing is well-established (McDonald and Schroeder, 2003; Johnson and Ward, 2005; Tatum et al., 2006; Harri et al., 2009). In a sensitivity analysis of economic factors, Horton et al. (2024) found minimal evidence to support extending the DOF of feedlot heifers. The authors reported that net returns for later-fed EPs compared to a baseline EP were poorest when fed cattle prices (live or dressed base) were low. This aligns with our study, where higher net returns frequently had higher live or dressed-fed cattle base prices. Although we observed that price adjustments from the premium and discount-based grid had an overall negative impact on net returns for later-fed EPs—which as previously discussed, disagreed with Horton et al. (2024)—we did find that increased net returns occurred more frequently (to some degree) with higher Choice-Select spreads, aligning with implications from Horton et al. (2024). We also note that beef marketing programs like Certified Angus Beef, which capitalize on cattle grading in the upper 2/3 Choice (along with additional requirements), were not simulated or incorporated in the QG grid and could provide additional revenue opportunities when grid marketing. Changes in premium and discount grids would likely impact the results. Specifically, heavyweight discounts have decreased substantially over past decades. While we used the industry average ($−16.50/cwt) which has been relatively stable, the USDA AMS has reported low-range discounts as little as $−5/cwt (LMIC). Reducing, or potentially removing the heavyweight discount would likely result in more favorable conditions for extending DOF.

Horton et al. (2024) reported that as FYP increased, net returns worsened for heifers fed to later EPs. This also aligns with our study, where higher net returns frequently had lower corn prices within each EP, relative to when returns were negative. Additionally, our findings indicate that corn price may be more critical than DMI, suggesting that the price of corn is a more important consideration than the amount cattle consume, relative to adding DOF. Furthermore, the relative importance of corn price and, to a lesser extent DMI, increased with each successive EP, highlighting the growing cost of feed as more days were added for both sale bases.

Late-day morbidity and mortality have become growing issues in the beef industry (Engler et al., 2014; Vogel et al., 2015; Smith et al., 2022a, 2022b), with risks increasing as cattle spend more time on feed. We parameterized health outcomes from trial data, as no other published data known by the authors report these risks for cattle-fed extended DOF in the specific timeframe evaluated. The incidence rates used for health outcomes reflect the trial’s study populations and may differ in other feedlot cattle. Vogel et al. (2015) reported 0.21% mortality during the last 30 DOF from observational data across all placement weight classes of feedlot steers. Extrapolating this value, it approximates to 0.07 mortalities per 1,000 animal-days-at-risk, slightly lower but comparable to our simulated mean of 0.09 mortalities per 1,000 animal-days. Despite changes in cattle populations and potential environmental or management differences between the studies, the similarity between these values supports our mortality parameterization. In the simulation model, the number of mortalities was the most important noneconomic variable influencing net return differences across both live and grid sale bases. Mortality was of higher relative importance within EP2 than EP3 or EP4, even though only 22.2% of simulations for EP2 had at least one mortality (out of 200 animals), compared to 39.6% and 53.0% for EP3 and EP4, respectively. While mortality was less frequent for EP2, its higher importance likely stems from less pen-weight gain (i.e., revenue) between EPs compared to EP3 and EP4, making it harder to compensate for the loss of a deceased animal. Consequently, while infrequent for EP2, when mortality does occur, it may be a more important driver of net return differences than other economic variables.

Other health outcomes were of lesser importance. The number of treatments was the lowest-ranked variable based on conditional importance scores, while removals had marginal to minimal importance. Several assumptions were required for the valuation (weight and price) of removed animals. The reasons for which feedlot cattle are culled are associated with weight (Horton et al., 2021), and we assumed removals in our model weighed similar to their pen mates. Horton et al. (2021) is the only known resource to provide a basis for estimating revenue from feedlot culls; therefore, adjusted cull cow prices were used. Although cattle populations differ between Horton et al. (2021) and those represented in the simulation model, substantial changes in our removal and revenue methodology would likely not alter interpretations of the simulation results due to the limited frequency of removals and their minimal impact on net returns.

Opportunity cost can vary in definition depending on the context. Some have characterized it as the cost of under- or overfeeding cattle relative to an “optimal” endpoint (Koontz et al., 2008); the cost of using a traditional marketing strategy instead of a profit-maximization rule (e.g., accounting for individual growth rate, where the value of marginal product equals the marginal cost; Maples et al., 2015); or the cost of not considering carcass characteristics in nonvalue-adjusted versus value-adjusted marketing (Poss et al., 2022). These references emphasize the importance of market timing for fed cattle. Similarly, our use of “net return differences” could be considered synonymous with opportunity cost, as it reflects the marginal costs or benefits of marketing at different EPs versus current standards. However, our definition of opportunity cost accounts for the cost of delayed marketing, where a longer wait period is required to receive revenue. Other than Horton et al. (2024) and Schmaltz et al. (2024), we have found no other peer-reviewed publications that discount the value of future dollars when evaluating differing DOF of feedlot cattle, despite this being standard in other economic applications (Rushton, 2007). A dollar today is more valuable than a future dollar because the current dollar can be invested to grow in value. Thus, future dollars must be discounted to reflect the cost of delaying revenue (Rushton, 2007). For example, if steers were marketed at EP1, the revenue could be invested, pay off operating loans, or be utilized elsewhere during the time spent waiting to market at later EPs.

Using a 5.85% fixed interest rate, Horton et al. (2024) estimated mean opportunity costs of approximately $4.13, $5.43, and $6.73 per animal for feedlot heifers marketed 21 d beyond a baseline EP at live fed cattle prices of $95, $125, and $155/cwt, respectively. Adjusted to 14-d increments to align with our simulations, these costs would be $2.75, $3.62, and $4.49 per animal. These opportunity costs for heifers were generally lower than what we found for steers, likely due to differences in weight (i.e., baseline revenue), interest rate, and fed cattle prices between the studies. Similarly, we also observed comparable opportunity costs between live and grid marketing bases. It is unclear how much producers consider opportunity cost when delaying marketing, and this likely varies among operations. In our evaluation, the interest rate was of marginal importance, while opportunity cost was minimal. Given the expected strong correlation between these variables in a nonconditional model, the higher ranking of interest rate suggests that opportunity cost may be more substantial when interest rates are higher. However, neither variable appeared to be a major influencer of net return differences compared to other economic variables, suggesting that opportunity cost may act more as a “penalty” that producers should consider rather than a key predictor of net return differences when feeding to later EPs.

It is important to recognize the extreme nature of the EPs in the trial data and our simulations, particularly EP4. Over 25% of simulated pens fed to EP4 had a mean HCW exceeding the threshold for heavyweight discounts, meaning more than half the animals were over 746 kg. The SD used to estimate the distribution of individual carcasses around the mean pen HCW was sourced from the trial data. Cattle in the trial were not sorted for pen uniformity, which could reduce carcass variability and help manage heavyweight and YG 4 and 5 carcasses (Koontz et al., 2008; Hilscher et al., 2015). However, a notable proportion of heavyweight carcasses should be expected when feeding to such extremes, regardless of how well sorting is implemented. By using trial data to parameterize the simulation model, we avoided relying on potentially outdated literature estimates that might require extrapolation beyond their source range. It is unclear how applicable growth or performance estimates from past literature are to contemporary cattle populations, especially when feeding well beyond norms. While feedlot cattle harvest weights may continue to increase, the steer population represented in our analyses will likely remain relevant for some time, while economic conditions like changing base prices and carcass discounts may benefit from adjustment in future years.

In our model, we assumed a passive, “price taker” approach by simulating random spot market outcomes without incorporating risk management strategies such as hedging or forward contracting. In practice, many feedlots actively manage risk through futures, options, or negotiated contracts, which can narrow the range of price outcomes and reduce both upside and downside volatility. Such risk management strategies may make extended feeding decisions less risky—effectively narrowing the distributions reported herein—potentially allowing producers to feed longer with greater confidence. Additionally, extending DOF under contractual delivery agreements may entail added costs and any hedges may need to be rolled forward as DOF are extended or fed cattle price risk may be fully exposed. However, this does not necessarily mean that the locations of the distributions would shift (i.e., approximate medians of each, the frequency of positive returns), and future research would be required to evaluate the implications of incorporating risk management strategies.

Beyond the considerations already discussed, additional limitations of the model should be acknowledged. Our simulation model framework addressed *what if* a pen with specific baseline characteristics (EP1) was alternatively fed to each of three later EPs, allowing comparison and characterization of distributions of marginal costs or benefits when extending DOF. The purpose of this framework was to describe the potential risks of feeding steers to later EPs so producers can make more informed decisions. In practice, producers must choose an EP in advance. Traditionally, the marketing window is refined when beginning to feed a beta-agonist like ractopamine HCl (Optaflexx; Elanco Animal Health), which was used in the trial and is labeled for 28 to 42 d of feeding. This has two main implications. First, once ractopamine is introduced, the marketing window is narrowed (i.e., changing to an alternative EP becomes more difficult when outside the labeled feeding duration). Lubabegron (Experior; Elanco Animal Health), a relatively new beta-agonist/-antagonist in the United States market, offers a wider feeding duration (14 to 91 d), potentially allowing for more flexibility in determining EPs. Lubabegron has also been shown to alter growth and carcass characteristics compared to ractopamine (McAtee et al., 2024), which may require additional considerations when feeding to later EPs. Second, there is a lag time between deciding the marketing window and actual harvest. Our results show that much of the risk and variability in net returns occurred due to fed cattle price changes when feeding to later EPs. Forecasting cattle prices is challenging, and the longer the time between decision and harvest, the greater the risk. Another limitation of the model is that it was constructed with pen-level inference rather than feedlot-level. Each simulation compared the optimal EP for the pen under specific conditions, without considering that marketing a pen opens space for new cattle. Feedlot-level opportunity costs may arise when determining the EP of a pen (i.e., what is optimal for a pen may not be so for the feedlot), which may vary depending on available pen space, as well as feeder cattle price, availability, and quality. Additionally, the model did not account for adverse events, such as extreme weather, that could occur between EPs and affect net returns.

Finally, no model can perfectly replicate biological or real-world systems, and models are limited by their assumptions. We used specific distributions, correlations, and parameters for stochastic simulation that, while evidence-based, must be interpreted with caveats regarding their applicability. Conditional random forest models were used to aid in identification of important variables in the simulation model and guide discussion. As with any model, the conditional variable importance scores reflect what was most important in the model, but this may not necessarily correspond to real-world importance. Moreover, many of the variables included in the random forests—such as actual fed cattle price differences, carcass grades, and future mortality—are unknown to producers at the time of EP decision-making. While machine learning models may offer viable approaches for EP management, that was not our objective. Future research may explore artificial intelligence or machine learning as tools for EP management in feedlot cattle; in some cases, such strategies may already be in place. Additionally, evaluating the environmental sustainability of feeding cattle to later EPs could be beneficial. While more time on feed likely increases resource use and carbon emissions per animal, producing more beef with fewer animals might offset this impact.

## Conclusion

We have effectively characterized the risks of feeding contemporary feedlot cattle to later EPs under recent pricing conditions across differing sale bases. The findings from this study provide critical insights into economic and performance considerations when extending DOF for contemporary feedlot steers under recent and varying market conditions. Feeding steers to later EPs introduces greater variability and risk in net returns, with grid marketing showing a higher potential for negative returns compared to live marketing, primarily due to the impact of carcass-based discounts. If marketing on a dressed-cash basis, feeding cattle to later endpoints could be advantageous, as net returns more frequently increased with extended feeding. However, this does not imply that dressed-cash marketing is more profitable than a grid approach, because baseline revenue comparisons between marketing strategies were not made. The most important factors in the simulation model were economic variables, with the differences in fed cattle prices received between later-fed EPs and EP1 being the most critical. While managing mortality risk is a relevant consideration when adding DOF, other cattle production and quality characteristics were of marginal to minimal importance. This implies that what may be considered an optimal EP based on weight and body composition of a pen, may not be the optimal economic EP, as timing and market conditions were more influential on net returns. Therefore, future research should recognize that the optimal EP for an animal or pen—based on body characteristics (e.g., weight, back-fat, carcass grade distribution) or marginal cost of gain—is likely a moving target, dependent on both economic conditions and risk. Ultimately, extending DOF and feeding steers to later EPs can be advantageous under favorable market conditions but carries inherent and increasing risks as feeding durations lengthen, with net return differences more often negative when marketing live or on a grid. Therefore, implementing appropriate risk management strategies may become increasingly important when feeding cattle to later EPs. All things considered, when selecting feedlot steer EPs and potentially extending DOF, producers aiming to optimize returns may need to prioritize market dynamics over traditional production metrics, while proactively managing risk.

## Supplementary Data

Supplementary data are available at *Journal of Animal Science* online.

skaf074_suppl_Supplementary_Tables_1-9_Figures_1-3

## Data Availability

The original contributions presented in the study are included in the article/Supplementary Material, further inquiries can be directed to the corresponding author.

## Abbreviations

95IPR: 95% inter-percentile range
AMS: Agricultural Marketing Service
bu: bushel
BW: body weight
CI: confidence interval
cwt: hundred-weight (45.4 kg or 100 lb)
DMI: dry matter intake
DOF: days-on-feed
EP: endpoint
FYP: feed and yardage price
GLMM: generalized linear mixed model
HCW: hot carcass weight
IQR: interquartile range
IP: implant program
LMM: linear mixed model
LMIC: Livestock Marketing Information Center
QG: USDA Quality Grade
USDA: United States Department of Agriculture
YG: USDA Yield Grade
