# Supplementary material for: Stochastic modeling of economic risk and net return distributions for feedlot steers marketed at alternative endpoints
Source: J Anim Sci. 2025 Mar 10;103:skaf074. doi: 10.1093/jas/skaf074 (PMC12124255; doi:10.1093/jas/skaf074)
Supplement: skaf074_suppl_Supplementary_Tables_1-9_Figures_1-3 [file skaf074_suppl_supplementary_tables_1-9_figures_1-3.pdf]

Supplemental Tables and Figures

Supplemental Table 1. Distributions of stochastic simulation results from parameterization of pricing variables that were used in economic models to evaluate distributions of net return differences when feeding steers to later endpoints (EPs)*

	Percentile								
Variable†	0.5	2.5	10	25	50	75	90	97.5	99.5
Corn price, $/bu	3.69	4.13	4.67	5.22	5.90	6.67	7.45	8.42	9.43
Feed and yardage price, $/ton DM	250.90	272.04	296.69	319.84	346.03	372.48	395.55	419.36	438.10
EP1 live fed price,‡ $/cwt	98.35	108.70	121.52	134.23	149.98	167.59	185.14	207.08	228.97
Difference from EP1 live price, $/cwt									
EP2	-9.58	-7.08	-4.49	-2.33	0.00	2.34	4.50	7.08	9.64
EP3	-13.61	-9.98	-6.34	-3.29	0.01	3.29	6.35	10.01	13.55
EP4	-15.21	-11.20	-7.09	-3.67	0.01	3.69	7.11	11.20	15.24
EP1 dressed fed base price,‡ $/cwt	156.10	172.55	192.88	213.07	238.06	266.01	293.87	328.70	363.45
Difference from EP1 dressed price , $/cwt									
EP2	-15.21	-11.23	-7.13	-3.69	0.00	3.72	7.15	11.23	15.30
EP3	-21.60	-15.83	-10.06	-5.22	0.01	5.22	10.08	15.89	21.51
EP4	-24.14	-17.77	-11.26	-5.82	0.01	5.86	11.29	17.78	24.19

* Stochastic simulation models (n = 360,000 simulations) parameterized animal performance, carcass characteristics, health, as well as economic variables to evaluate differences in net returns when feeding steers to later endpoints under variable conditions

† Abbreviations: bu = bushel (25.4 kg or 56 lb); US ton = 907 kg or 2,000 lb; DM = dry matter; cwt = hundred-weight (45.4 kg or 100 lb)

‡ Later-fed EPs had nominally identical distributions of fed cattle prices

Supplemental Table 2. Distributions of stochastic simulation results from parameterization of steer performance variables that were used in the economic model to evaluate distributions of net return differences when feeding steers to later endpoints (EPs)*

	Percentile								
Variable	0.5	2.5	10	25	50	75	90	97.5	99.5
Dry matter intake, kg/d	8.98	9.34	9.73	10.09	10.48	10.87	11.22	11.62	11.97
Live weight gain, kg/d	1.17	1.21	1.25	1.28	1.32	1.37	1.40	1.45	1.48
Carcass weight gain, kg/d	0.89	0.92	0.94	0.97	1.00	1.03	1.05	1.08	1.11
Final live weight, kg									
EP1	637	644	652	659	667	674	681	689	696
EP2	656	663	671	678	685	693	700	708	715
EP3	674	681	689	696	704	712	719	727	734
EP4	692	699	707	715	722	730	737	745	753
Hot carcass weight, kg									
EP1	408	414	420	425	431	437	442	448	454
EP2	422	428	434	439	445	451	456	462	468
EP3	436	441	447	453	459	465	470	476	482
EP4	450	455	461	467	473	479	484	491	496

* Stochastic simulation models (n = 360,000 simulations) parameterized animal performance, carcass characteristics, health, as well as economic variables to evaluate differences in net returns when feeding steers to later endpoints under variable conditions

Supplemental Table 3. Distributions of stochastic simulation results from parameterization of carcass characteristic variables that were used in economic models to evaluate distributions of net return differences when feeding steers to later endpoints (EPs)*

	Percentile								
Variable†	0.5	2.5	10	25	50	75	90	97.5	99.5
Yield Grade 1, %									
EP1	4.0	5.2	6.5	7.8	9.2	10.9	12.8	15.9	19.9
EP2	2.4	3.2	4.1	5.1	6.2	7.5	9.1	11.6	14.7
EP3	1.8	2.4	3.1	3.8	4.6	5.7	6.9	8.8	11.4
EP4	1.0	1.4	1.8	2.2	2.7	3.3	4.0	5.2	6.7
Yield Grade 2, %									
EP1	22.3	26.7	30.9	34.0	37.1	40.0	42.7	45.8	48.6
EP2	15.3	19.1	22.9	26.2	29.8	33.4	36.8	41.0	44.9
EP3	12.0	15.1	18.5	21.4	24.7	28.2	31.7	36.2	40.7
EP4	7.3	9.3	11.6	13.8	16.3	19.1	22.1	26.3	31.1
Yield Grade 3, %									
EP1	25.5	29.7	33.5	36.1	38.4	40.4	42.0	43.5	44.7
EP2	31.1	35.2	38.5	40.6	42.2	43.1	43.9	44.6	45.2
EP3	34.4	38.0	40.7	42.0	42.9	43.6	44.2	44.8	45.3
EP4	28.4	32.7	36.4	39.0	41.1	42.6	43.6	44.5	45.3
Yield Grade 4, %									
EP1	5.7	7.3	9.2	10.7	12.5	14.5	16.7	20.0	24.1
EP2	8.0	10.1	12.7	14.9	17.5	20.4	23.4	27.5	31.9
EP3	10.4	13.0	16.1	18.7	21.7	24.9	28.1	32.2	36.5
EP4	16.5	20.2	24.1	27.2	30.5	33.8	36.7	40.0	43.0
Yield Grade 5, %									
EP1	1.1	1.5	1.9	2.2	2.7	3.2	3.9	4.9	6.4
EP2	1.6	2.1	2.7	3.3	4.1	5.0	6.1	7.8	10.1
EP3	2.1	2.8	3.6	4.4	5.4	6.6	8.0	10.2	13.1
EP4	3.7	4.9	6.3	7.6	9.3	11.2	13.4	16.8	21.2

* Stochastic simulation models (n = 360,000 simulations) parameterized animal performance, carcass characteristics, health, as well as economic variables to evaluate differences in net returns when feeding steers to later endpoints under variable conditions

† Yield and Quality Grade values are percentage values that were back-transformed after simulation on the logit scale using a proportional odds model framework

Supplemental Table 3. Continued

Variable[†]	Percentile								
	0.5	2.5	10	25	50	75	90	97.5	99.5
Prime, %									
EP1	0.4	0.8	1.2	1.7	2.5	3.5	5.0	7.9	12.8
EP2	0.6	1.0	1.6	2.3	3.4	4.8	6.8	10.7	17.1
EP3	0.7	1.2	2.0	2.9	4.1	5.9	8.3	12.8	20.2
EP4	0.9	1.5	2.4	3.5	5.0	7.1	10.0	15.3	23.8
Choice, %									
EP1	28.8	40.3	51.6	59.7	66.9	72.8	77.1	80.2	82.1
EP2	34.9	47.2	58.4	65.9	72.2	76.8	79.4	81.3	82.7
EP3	39.6	52.1	62.9	69.7	75.0	78.5	80.3	81.8	82.9
EP4	44.3	56.8	66.8	72.8	77.1	79.5	80.9	82.0	83.0
Select, %									
EP1	7.2	11.7	17.3	22.9	29.6	37.1	45.1	55.7	65.6
EP2	5.3	8.6	13.1	17.7	23.6	30.6	38.4	49.3	60.5
EP3	4.3	7.1	11.0	14.9	20.1	26.5	33.8	44.5	56.3
EP4	3.6	5.9	9.1	12.5	17.1	22.8	29.5	39.8	51.9
Sub-Select, %									
EP1	0.2	0.3	0.4	0.7	1.0	1.5	2.1	3.6	6.3
EP2	0.1	0.2	0.3	0.5	0.7	1.1	1.6	2.7	4.8
EP3	0.1	0.2	0.3	0.4	0.6	0.9	1.3	2.2	3.9
EP4	0.1	0.1	0.2	0.3	0.5	0.7	1.1	1.8	3.2
Heavyweight carcasses,[‡] %									
EP1	3.9	5.2	7.1	9.2	12.0	15.3	18.9	23.4	27.9
EP2	8.0	10.3	13.4	16.6	20.8	25.5	30.2	35.8	41.3
EP3	14.8	18.3	22.7	27.2	32.6	38.3	43.8	50.1	55.8
EP4	24.4	29.2	34.9	40.3	46.5	52.7	58.3	64.4	69.7

[†] Yield and Quality Grade values are percentage values that were back-transformed after simulation on the logit scale using a proportional odds model framework

[‡] Heavyweight carcasses were those greater than or equal to 746 kg (1,050 lb)

Supplemental Table 4. Distributions of stochastic simulation results from parameterization of health variables that were used in economic models to evaluate distributions of net return differences when feeding steers to later endpoints (EPs)[*,†]

	Percentile								
Variable	0.5	2.5	10	25	50	75	90	97.5	99.5
Morbidity, n/pen									
EP2	0	0	0	0	1	1	2	3	4
EP3	0	0	0	1	1	2	3	5	6
EP4	0	0	1	1	2	3	5	6	7
Removals, n/pen									
EP2	0	0	0	0	0	1	1	2	3
EP3	0	0	0	0	1	2	2	3	4
EP4	0	0	0	1	1	2	3	4	5
Mortality, n/pen									
EP2	0	0	0	0	0	0	1	2	2
EP3	0	0	0	0	0	1	1	2	3
EP4	0	0	0	0	1	1	2	3	4

[*] Stochastic simulation models (n = 360,000 simulations) parameterized animal performance, carcass characteristics, health, as well as economic variables to evaluate differences in net returns when feeding steers to later endpoints under variable conditions

[†] The number of morbidities, removals, and mortalities were simulated with an initial population of 200 animals in the pen (i.e., values are the counts of events per 200 animals given the animal-time at risk for each EP)

Supplemental Table 5. Tabular percentile values of the distributions of net return differences comparing later fed endpoints (EPs) to EP1 from a stochastic simulation model of beef feedlot steers marketed on a dressed-cash basis*

Net return difference from EP1, $/animal	Percentile														
	0.5	2.5	5	10	20	30	40	50	60	70	80	90	95	97.5	99.5
EP2	-136.39	-100.36	-83.13	-63.27	-39.72	-22.96	-8.45	5.27	19.25	34.46	52.94	79.50	102.46	123.30	167.99
EP3	-192.28	-142.63	-117.72	-89.49	-55.47	-30.81	-9.61	10.40	31.08	53.88	81.16	120.82	155.30	187.01	252.97
EP4	-218.54	-161.74	-133.52	-101.06	-61.76	-33.06	-8.18	15.59	40.02	66.87	99.32	147.15	189.39	227.15	308.49

* Stochastic simulation models (n = 360,000 simulations) parameterized animal performance, carcass characteristics, health, as well as economic variables to evaluate differences in net returns when feeding steers to later endpoints under variable conditions

Supplemental Table 6. Descriptive statistics of economic and cattle performance variables which were included in conditional random forest models, categorized by net return difference categories for later fed endpoints (EPs) from a stochastic simulation model of feedlot steers marketed live or dressed with a premium and discount based grid*,†

	Live price difference from EP1, $/cwt					Dressed base price difference from EP1, $/cwt				
Net return difference category	Mean	SD	Med	IQR	95IPR	Mean	SD	Med	IQR	95IPR
Endpoint 2										
< $-25/animal	-3.56	2.14	-3.16	-4.71 to -2.02	-8.71 to -0.45	-5.34	3.53	-4.78	-7.30 to -2.81	-13.70 to -0.01
$-25 to $-5/animal	-0.56	0.93	-0.55	-1.17 to 0.06	-2.43 to 1.21	-0.66	1.77	-0.59	-1.82 to 0.56	-4.31 to 2.64
$-5 to $5/animal	0.39	0.86	0.41	-0.17 to 0.96	-1.35 to 2.01	0.75	1.67	0.82	-0.34 to 1.91	-2.69 to 3.84
$5 to $25/animal	1.30	0.91	1.31	0.70 to 1.91	-0.51 to 3.03	2.10	1.73	2.15	0.97 to 3.30	-1.47 to 5.34
> $25/animal	4.14	2.04	3.77	2.69 to 5.21	1.14 to 9.10	6.49	3.35	6.00	4.18 to 8.30	1.22 to 14.46
Endpoint 3										
< $-25/animal	-4.27	3.24	-3.78	-6.09 to -1.94	-11.87 to 0.66	-5.92	5.41	-5.19	-9.04 to -2.03	-18.40 to 2.58
$-25 to $-5/animal	-0.18	1.56	-0.12	-1.19 to 0.89	-3.43 to 2.71	0.75	2.86	0.89	-1.07 to 2.73	-5.29 to 5.99
$-5 to $5/animal	0.72	1.53	0.80	-0.26 to 1.77	-2.44 to 3.53	2.08	2.82	2.23	0.28 to 4.02	-3.85 to 7.26
$5 to $25/animal	1.59	1.55	1.65	0.59 to 2.66	-1.61 to 4.47	3.32	2.82	3.46	1.50 to 5.27	-2.57 to 8.51
> $25/animal	5.34	3.07	4.89	3.21 to 7.01	0.52 to 12.57	8.98	4.95	8.40	5.59 to 11.73	0.80 to 20.40
Endpoint 4										
< $-25/animal	-4.32	3.82	-3.82	-6.52 to -1.58	-13.13 to 1.78	-4.92	6.58	-4.19	-8.86 to -0.21	-19.80 to 5.84
$-25 to $-5/animal	0.14	2.15	0.24	-1.24 to 1.63	-4.32 to 4.08	2.99	3.85	3.19	0.51 to 5.70	-5.15 to 9.93
$-5 to $5/animal	0.99	2.14	1.08	-0.40 to 2.46	-3.50 to 4.95	4.19	3.81	4.40	1.78 to 6.85	-3.83 to 11.08
$5 to $25/animal	1.80	2.14	1.89	0.44 to 3.27	-2.66 to 5.72	5.33	3.77	5.51	2.93 to 7.96	-2.49 to 12.27
> $25/animal	5.81	3.62	5.40	3.34 to 7.86	-0.28 to 14.11	11.05	5.76	10.59	7.21 to 14.39	0.90 to 23.92

* Stochastic simulation models (n = 360,000 simulations) parameterized animal performance, carcass characteristics, health, as well as economic variables to evaluate differences in net returns when feeding steers to later endpoints under variable conditions

† Abbreviations: Med = median; IQR = interquartile range (25th to 75th percentile); 95IPR = 95th inter-percentile range (2.5th to 97.5th percentile); cwt = hundred-weight (45.4 kg or 100 lb); bu = bushel (25.4 kg or 56 lb)

Supplemental Table 6. Continued

	Live fed cattle price, $/cwt					Dressed fed cattle base price, $/cwt				
Net return difference category	Mean	SD	Med	IQR	95IPR	Mean	SD	Med	IQR	95IPR
Endpoint 2										
< $-25/animal	147.82	24.36	145.79	130.57 to 162.88	105.87 to 201.11	233.48	38.42	230.35	206.22 to 257.22	167.40 to 317.50
$-25 to $-5/animal	147.34	23.87	145.46	130.46 to 162.28	106.18 to 199.44	234.29	37.59	231.29	207.89 to 257.50	169.60 to 316.68
$-5 to $5/animal	148.85	23.85	147.00	132.13 to 163.38	107.90 to 201.00	236.98	37.48	234.16	210.13 to 260.33	172.77 to 318.46
$5 to $25/animal	151.06	23.80	149.11	134.13 to 165.80	110.23 to 203.32	240.59	37.61	237.39	213.94 to 263.65	176.02 to 323.28
> $25/animal	161.23	25.32	159.17	143.32 to 176.89	117.72 to 216.73	257.10	40.13	253.86	228.64 to 281.82	188.06 to 344.98
Endpoint 3										
< $-25/animal	145.73	23.73	143.81	128.93 to 160.35	104.87 to 197.70	230.68	37.19	227.64	204.35 to 253.60	166.69 to 311.98
$-25 to $-5/animal	148.18	23.24	146.17	131.87 to 162.67	108.30 to 199.05	237.33	36.71	234.26	211.30 to 260.24	174.35 to 317.01
$-5 to $5/animal	149.74	23.39	147.71	133.18 to 164.36	109.96 to 200.62	240.26	36.51	237.27	214.52 to 262.92	177.16 to 320.48
$5 to $25/animal	151.53	23.32	149.74	135.10 to 165.89	111.42 to 202.66	243.59	36.28	240.66	218.16 to 265.82	181.11 to 323.00
> $25/animal	162.43	25.05	160.39	144.72 to 177.90	119.39 to 217.50	261.23	39.66	258.00	233.14 to 285.66	193.26 to 348.37
Endpoint 4										
< $-25/animal	144.48	23.15	142.61	128.15 to 158.81	104.65 to 194.89	230.42	36.26	227.62	204.89 to 252.85	167.60 to 309.20
$-25 to $-5/animal	149.11	22.87	147.31	133.13 to 163.02	109.72 to 199.09	243.48	35.57	240.61	218.28 to 265.48	182.23 to 321.80
$-5 to $5/animal	150.78	22.55	148.98	135.00 to 164.66	112.19 to 200.38	246.45	35.31	243.11	221.73 to 268.34	186.03 to 324.23
$5 to $25/animal	152.35	22.69	150.33	136.46 to 166.20	113.41 to 202.39	250.00	35.60	247.13	224.82 to 272.32	188.37 to 327.10
> $25/animal	164.16	24.85	162.15	146.53 to 179.55	121.48 to 218.61	268.86	39.13	265.79	241.22 to 292.90	201.61 to 354.71

Supplemental Table 6. Continued

	Corn price, $/bu (live sale basis)					Corn price, $/bu (grid sale basis)				
Net return difference category	Mean	SD	Med	IQR	95IPR	Mean	SD	Med	IQR	95IPR
Endpoint 2										
< $-25/animal	6.13	1.12	6.03	5.34 to 6.82	4.23 to 8.59	6.12	1.12	6.02	5.33 to 6.80	4.22 to 8.58
$-25 to $-5/animal	6.01	1.09	5.92	5.24 to 6.68	4.15 to 8.41	6.01	1.10	5.92	5.23 to 6.68	4.14 to 8.41
$-5 to $5/animal	5.98	1.09	5.88	5.21 to 6.65	4.13 to 8.37	5.98	1.09	5.88	5.22 to 6.65	4.13 to 8.39
$5 to $25/animal	5.95	1.08	5.85	5.18 to 6.61	4.10 to 8.32	5.94	1.08	5.84	5.17 to 6.60	4.10 to 8.30
> $25/animal	5.86	1.07	5.76	5.10 to 6.51	4.04 to 8.23	5.86	1.07	5.76	5.11 to 6.52	4.04 to 8.24
Endpoint 3										
< $-25/animal	6.16	1.12	6.06	5.37 to 6.84	4.25 to 8.62	6.13	1.11	6.03	5.34 to 6.81	4.23 to 8.58
$-25 to $-5/animal	5.99	1.09	5.90	5.22 to 6.65	4.13 to 8.37	5.98	1.09	5.88	5.21 to 6.64	4.11 to 8.37
$-5 to $5/animal	5.96	1.09	5.87	5.19 to 6.63	4.12 to 8.38	5.95	1.08	5.85	5.18 to 6.63	4.13 to 8.30
$5 to $25/animal	5.93	1.07	5.83	5.17 to 6.60	4.11 to 8.30	5.91	1.07	5.81	5.16 to 6.56	4.09 to 8.28
> $25/animal	5.81	1.06	5.72	5.07 to 6.46	4.02 to 8.15	5.81	1.06	5.71	5.06 to 6.46	4.02 to 8.15
Endpoint 4										
< $-25/animal	6.19	1.12	6.09	5.40 to 6.87	4.28 to 8.64	6.13	1.11	6.03	5.34 to 6.81	4.23 to 8.56
$-25 to $-5/animal	5.97	1.07	5.88	5.21 to 6.63	4.12 to 8.30	5.89	1.07	5.80	5.14 to 6.54	4.08 to 8.24
$-5 to $5/animal	5.94	1.06	5.85	5.20 to 6.59	4.11 to 8.24	5.88	1.07	5.78	5.12 to 6.53	4.06 to 8.21
$5 to $25/animal	5.90	1.07	5.80	5.15 to 6.55	4.10 to 8.26	5.85	1.06	5.75	5.10 to 6.49	4.03 to 8.24
> $25/animal	5.76	1.05	5.67	5.02 to 6.40	3.99 to 8.09	5.74	1.04	5.64	5.00 to 6.37	3.98 to 8.04

Supplemental Table 6. Continued

	Opportunity cost, $/animal (live sale basis)					Opportunity cost, $/animal (grid sale basis)				
Net return difference category	Mean	SD	Med	IQR	95IPR	Mean	SD	Med	IQR	95IPR
Endpoint 2										
< $-25/animal	6.02	1.73	5.87	4.60 to 7.21	3.33 to 9.67	5.95	1.74	5.79	4.54 to 7.13	3.26 to 9.63
$-25 to $-5/animal	5.83	1.68	5.68	4.45 to 6.99	3.25 to 9.37	5.79	1.70	5.62	4.41 to 6.95	3.19 to 9.41
$-5 to $5/animal	5.85	1.69	5.70	4.46 to 7.03	3.25 to 9.41	5.81	1.70	5.65	4.43 to 6.99	3.19 to 9.42
$5 to $25/animal	5.90	1.70	5.74	4.52 to 7.08	3.30 to 9.50	5.88	1.71	5.72	4.49 to 7.05	3.25 to 9.55
> $25/animal	6.16	1.77	5.99	4.70 to 7.38	3.46 to 9.90	6.13	1.79	5.96	4.68 to 7.36	3.40 to 9.94
Endpoint 3										
< $-25/animal	11.93	3.44	11.66	9.13 to 14.30	6.60 to 19.21	11.78	3.44	11.47	8.99 to 14.12	6.45 to 19.11
$-25 to $-5/animal	11.70	3.38	11.39	8.93 to 14.04	6.51 to 18.80	11.66	3.42	11.31	8.89 to 14.01	6.40 to 18.91
$-5 to $5/animal	11.74	3.40	11.43	8.93 to 14.09	6.53 to 18.95	11.71	3.42	11.39	8.92 to 14.10	6.47 to 18.94
$5 to $25/animal	11.81	3.41	11.50	9.02 to 14.22	6.61 to 18.94	11.81	3.44	11.47	9.01 to 14.22	6.55 to 19.11
> $25/animal	12.28	3.53	11.93	9.39 to 14.74	6.91 to 19.76	12.31	3.58	11.95	9.40 to 14.77	6.86 to 19.95
Endpoint 4										
< $-25/animal	17.78	5.12	17.37	13.61 to 21.31	9.83 to 28.60	17.57	5.13	17.12	13.41 to 21.07	9.63 to 28.45
$-25 to $-5/animal	17.64	5.11	17.20	13.44 to 21.16	9.79 to 28.41	17.71	5.16	17.18	13.49 to 21.26	9.86 to 28.62
$-5 to $5/animal	17.74	5.09	17.30	13.57 to 21.26	9.92 to 28.44	17.75	5.19	17.26	13.50 to 21.27	9.91 to 29.01
$5 to $25/animal	17.75	5.12	17.28	13.54 to 21.32	9.99 to 28.53	17.95	5.23	17.45	13.67 to 21.53	10.05 to 29.23
> $25/animal	18.52	5.33	17.98	14.14 to 22.23	10.45 to 29.79	18.80	5.45	18.24	14.32 to 22.60	10.53 to 30.37

Supplemental Table 6. Continued

	Dry matter intake, kg/animal/d (live sale basis)					Dry matter intake, kg/animal/d (grid sale basis)				
Net return difference category	Mean	SD	Med	IQR	95IPR	Mean	SD	Med	IQR	95IPR
Endpoint 2										
< $-25/animal	10.51	0.58	10.51	10.13 to 10.91	9.37 to 11.65	10.51	0.58	10.51	10.12 to 10.90	9.37 to 11.65
$-25 to $-5/animal	10.48	0.58	10.48	10.09 to 10.87	9.34 to 11.61	10.48	0.58	10.48	10.09 to 10.87	9.33 to 11.62
$-5 to $5/animal	10.47	0.58	10.47	10.08 to 10.86	9.33 to 11.62	10.47	0.58	10.47	10.08 to 10.86	9.34 to 11.60
$5 to $25/animal	10.46	0.58	10.46	10.07 to 10.85	9.34 to 11.60	10.47	0.58	10.47	10.08 to 10.85	9.35 to 11.60
> $25/animal	10.44	0.58	10.44	10.05 to 10.83	9.30 to 11.57	10.44	0.58	10.44	10.05 to 10.83	9.30 to 11.57
Endpoint 3										
< $-25/animal	10.52	0.58	10.52	10.13 to 10.91	9.38 to 11.66	10.51	0.58	10.51	10.13 to 10.90	9.37 to 11.65
$-25 to $-5/animal	10.47	0.58	10.47	10.09 to 10.86	9.34 to 11.60	10.46	0.58	10.46	10.07 to 10.86	9.33 to 11.60
$-5 to $5/animal	10.47	0.58	10.47	10.08 to 10.86	9.34 to 11.60	10.47	0.58	10.47	10.08 to 10.86	9.33 to 11.59
$5 to $25/animal	10.45	0.58	10.45	10.06 to 10.84	9.33 to 11.58	10.45	0.58	10.45	10.06 to 10.84	9.33 to 11.58
> $25/animal	10.43	0.58	10.43	10.04 to 10.82	9.30 to 11.57	10.43	0.58	10.43	10.04 to 10.82	9.30 to 11.57
Endpoint 4										
< $-25/animal	10.53	0.58	10.53	10.14 to 10.92	9.39 to 11.66	10.51	0.58	10.51	10.12 to 10.90	9.38 to 11.65
$-25 to $-5/animal	10.47	0.57	10.47	10.08 to 10.85	9.34 to 11.58	10.45	0.58	10.45	10.06 to 10.84	9.32 to 11.58
$-5 to $5/animal	10.46	0.58	10.46	10.08 to 10.85	9.33 to 11.58	10.44	0.59	10.44	10.05 to 10.84	9.30 to 11.58
$5 to $25/animal	10.45	0.58	10.45	10.05 to 10.84	9.31 to 11.58	10.43	0.58	10.44	10.04 to 10.82	9.28 to 11.56
> $25/animal	10.42	0.58	10.41	10.03 to 10.80	9.28 to 11.55	10.41	0.58	10.41	10.02 to 10.80	9.28 to 11.54

Supplemental Table 6. Continued

	Final live weight, kg/animal					Hot carcass weight, kg/carcass				
Net return difference category	Mean	SD	Med	IQR	95IPR	Mean	SD	Med	IQR	95IPR
Endpoint 2										
< $-25/animal	685	11.4	685	678 to 693	663 to 708	445	8.8	446	440 to 451	428 to 463
$-25 to $-5/animal	685	11.5	685	677 to 693	663 to 707	445	8.8	445	439 to 451	427 to 462
$-5 to $5/animal	685	11.5	685	677 to 693	662 to 708	445	8.9	445	439 to 451	427 to 462
$5 to $25/animal	685	11.5	685	677 to 693	663 to 708	444	8.9	445	439 to 450	427 to 462
> $25/animal	686	11.4	685	678 to 693	663 to 708	444	8.8	444	438 to 450	427 to 462
Endpoint 3										
< $-25/animal	704	11.6	704	696 to 712	681 to 726	459	8.9	459	453 to 465	442 to 477
$-25 to $-5/animal	704	11.6	704	696 to 711	681 to 726	459	8.9	458	453 to 464	441 to 476
$-5 to $5/animal	704	11.5	704	696 to 711	681 to 726	459	8.8	459	453 to 464	441 to 476
$5 to $25/animal	704	11.5	704	696 to 712	681 to 726	458	8.9	458	452 to 464	441 to 476
> $25/animal	704	11.5	704	696 to 712	681 to 727	458	9.0	458	452 to 464	441 to 476
Endpoint 4										
< $-25/animal	722	11.7	722	714 to 730	699 to 745	473	9.0	473	467 to 479	456 to 491
$-25 to $-5/animal	722	11.8	722	714 to 730	699 to 745	472	9.0	472	466 to 478	454 to 490
$-5 to $5/animal	722	11.8	722	714 to 730	699 to 745	472	9.1	472	466 to 478	454 to 490
$5 to $25/animal	722	11.7	722	714 to 730	699 to 745	472	9.0	472	466 to 478	455 to 490
> $25/animal	723	11.7	723	715 to 731	700 to 746	472	9.1	472	466 to 478	454 to 490

Supplemental Table 6. Continued

	Live weight gain, kg/animal/d					Carcass weight gain, kg/carcass/d				
Net return difference category	Mean	SD	Med	IQR	95IPR	Mean	SD	Med	IQR	95IPR
Endpoint 2										
< $-25/animal	1.32	0.06	1.32	1.28 to 1.36	1.20 to 1.44	1.00	0.04	1.00	0.97 to 1.02	0.91 to 1.08
$-25 to $-5/animal	1.32	0.06	1.32	1.28 to 1.37	1.21 to 1.44	1.00	0.04	1.00	0.97 to 1.03	0.92 to 1.08
$-5 to $5/animal	1.32	0.06	1.32	1.28 to 1.37	1.20 to 1.45	1.00	0.04	1.00	0.97 to 1.03	0.92 to 1.08
$5 to $25/animal	1.33	0.06	1.33	1.28 to 1.37	1.21 to 1.45	1.00	0.04	1.00	0.97 to 1.03	0.92 to 1.08
> $25/animal	1.33	0.06	1.33	1.29 to 1.37	1.21 to 1.45	1.00	0.04	1.00	0.97 to 1.03	0.92 to 1.08
Endpoint 3										
< $-25/animal	1.32	0.06	1.32	1.28 to 1.36	1.20 to 1.44	1.00	0.04	1.00	0.97 to 1.02	0.91 to 1.08
$-25 to $-5/animal	1.32	0.06	1.32	1.28 to 1.37	1.21 to 1.45	1.00	0.04	1.00	0.97 to 1.03	0.92 to 1.08
$-5 to $5/animal	1.33	0.06	1.33	1.28 to 1.37	1.21 to 1.44	1.00	0.04	1.00	0.97 to 1.03	0.92 to 1.08
$5 to $25/animal	1.33	0.06	1.33	1.29 to 1.37	1.21 to 1.45	1.00	0.04	1.00	0.97 to 1.03	0.92 to 1.08
> $25/animal	1.33	0.06	1.33	1.29 to 1.37	1.21 to 1.45	1.00	0.04	1.00	0.97 to 1.03	0.92 to 1.08
Endpoint 4										
< $-25/animal	1.32	0.06	1.32	1.28 to 1.36	1.20 to 1.44	1.00	0.04	1.00	0.97 to 1.02	0.91 to 1.08
$-25 to $-5/animal	1.33	0.06	1.32	1.28 to 1.37	1.21 to 1.45	1.00	0.04	1.00	0.97 to 1.03	0.92 to 1.08
$-5 to $5/animal	1.33	0.06	1.33	1.29 to 1.37	1.21 to 1.45	1.00	0.04	1.00	0.97 to 1.03	0.92 to 1.08
$5 to $25/animal	1.33	0.06	1.33	1.29 to 1.37	1.21 to 1.45	1.00	0.04	1.00	0.97 to 1.03	0.92 to 1.08
> $25/animal	1.33	0.06	1.33	1.29 to 1.37	1.21 to 1.45	1.00	0.04	1.00	0.97 to 1.03	0.92 to 1.08

Supplemental Table 6. Continued

	Mortality, n/pen (live sale basis)					Mortality, n/pen (grid sale basis)				
Net return difference category	Mean	SD	Med	IQR	95IPR	Mean	SD	Med	IQR	95IPR
Endpoint 2										
< $-25/animal	0.31	0.55	0	0 to 1	0 to 2	0.30	0.55	0	0 to 1	0 to 2
$-25 to $-5/animal	0.25	0.50	0	0 to 0	0 to 2	0.25	0.50	0	0 to 0	0 to 2
$-5 to $5/animal	0.24	0.49	0	0 to 0	0 to 1	0.24	0.49	0	0 to 0	0 to 1
$5 to $25/animal	0.22	0.47	0	0 to 0	0 to 1	0.22	0.47	0	0 to 0	0 to 1
> $25/animal	0.19	0.44	0	0 to 0	0 to 1	0.19	0.44	0	0 to 0	0 to 1
Endpoint 3										
< $-25/animal	0.57	0.75	0	0 to 1	0 to 2	0.56	0.74	0	0 to 1	0 to 2
$-25 to $-5/animal	0.50	0.70	0	0 to 1	0 to 2	0.49	0.69	0	0 to 1	0 to 2
$-5 to $5/animal	0.49	0.69	0	0 to 1	0 to 2	0.48	0.69	0	0 to 1	0 to 2
$5 to $25/animal	0.47	0.68	0	0 to 1	0 to 2	0.47	0.68	0	0 to 1	0 to 2
> $25/animal	0.42	0.65	0	0 to 1	0 to 2	0.42	0.65	0	0 to 1	0 to 2
Endpoint 4										
< $-25/animal	0.84	0.91	1	0 to 1	0 to 3	0.81	0.89	1	0 to 1	0 to 3
$-25 to $-5/animal	0.73	0.85	1	0 to 1	0 to 3	0.71	0.83	1	0 to 1	0 to 3
$-5 to $5/animal	0.73	0.84	1	0 to 1	0 to 3	0.70	0.82	1	0 to 1	0 to 3
$5 to $25/animal	0.71	0.83	1	0 to 1	0 to 3	0.68	0.82	0	0 to 1	0 to 3
> $25/animal	0.65	0.80	0	0 to 1	0 to 3	0.64	0.79	0	0 to 1	0 to 3

Supplemental Table 6. Continued

	Removals, n/pen (live sale basis)					Removals, n/pen (grid sale basis)				
Net return difference category	Mean	SD	Med	IQR	95IPR	Mean	SD	Med	IQR	95IPR
Endpoint 2										
< $-25/animal	0.55	0.74	0	0 to 1	0 to 2	0.55	0.74	0	0 to 1	0 to 2
$-25 to $-5/animal	0.51	0.72	0	0 to 1	0 to 2	0.51	0.71	0	0 to 1	0 to 2
$-5 to $5/animal	0.50	0.71	0	0 to 1	0 to 2	0.51	0.71	0	0 to 1	0 to 2
$5 to $25/animal	0.49	0.70	0	0 to 1	0 to 2	0.50	0.70	0	0 to 1	0 to 2
> $25/animal	0.47	0.69	0	0 to 1	0 to 2	0.47	0.69	0	0 to 1	0 to 2
Endpoint 3										
< $-25/animal	1.07	1.03	1	0 to 2	0 to 3	1.06	1.02	1	0 to 2	0 to 3
$-25 to $-5/animal	1.01	1.01	1	0 to 2	0 to 3	1.02	1.02	1	0 to 2	0 to 3
$-5 to $5/animal	1.01	1.00	1	0 to 2	0 to 3	1.00	1.00	1	0 to 2	0 to 3
$5 to $25/animal	1.00	1.01	1	0 to 2	0 to 3	0.99	0.99	1	0 to 2	0 to 3
> $25/animal	0.96	0.98	1	0 to 2	0 to 3	0.96	0.98	1	0 to 2	0 to 3
Endpoint 4										
< $-25/animal	1.58	1.26	1	1 to 2	0 to 4	1.56	1.25	1	1 to 2	0 to 4
$-25 to $-5/animal	1.52	1.24	1	1 to 2	0 to 4	1.50	1.21	1	1 to 2	0 to 4
$-5 to $5/animal	1.50	1.22	1	1 to 2	0 to 4	1.49	1.23	1	1 to 2	0 to 4
$5 to $25/animal	1.49	1.21	1	1 to 2	0 to 4	1.48	1.22	1	1 to 2	0 to 4
> $25/animal	1.46	1.21	1	1 to 2	0 to 4	1.45	1.20	1	1 to 2	0 to 4

Supplemental Table 6. Continued

Net return difference category	Treatments, n/pen (live sale basis)					Treatments, n/pen (grid sale basis)				
	Mean	SD	Med	IQR	95IPR	Mean	SD	Med	IQR	95IPR
Endpoint 2										
< $-25/animal	0.83	0.91	1	0 to 1	0 to 3	0.82	0.91	1	0 to 1	0 to 3
$-25 to $-5/animal	0.82	0.91	1	0 to 1	0 to 3	0.82	0.91	1	0 to 1	0 to 3
$-5 to $5/animal	0.82	0.90	1	0 to 1	0 to 3	0.82	0.90	1	0 to 1	0 to 3
$5 to $25/animal	0.81	0.90	1	0 to 1	0 to 3	0.82	0.91	1	0 to 1	0 to 3
> $25/animal	0.82	0.91	1	0 to 1	0 to 3	0.82	0.91	1	0 to 1	0 to 3
Endpoint 3										
< $-25/animal	1.64	1.28	1	1 to 2	0 to 5	1.64	1.28	1	1 to 2	0 to 5
$-25 to $-5/animal	1.65	1.29	1	1 to 2	0 to 5	1.65	1.29	1	1 to 2	0 to 5
$-5 to $5/animal	1.65	1.27	1	1 to 2	0 to 5	1.64	1.27	1	1 to 2	0 to 5
$5 to $25/animal	1.64	1.28	1	1 to 2	0 to 5	1.64	1.28	1	1 to 2	0 to 4
> $25/animal	1.64	1.28	1	1 to 2	0 to 5	1.64	1.28	1	1 to 2	0 to 5
Endpoint 4										
< $-25/animal	2.46	1.57	2	1 to 3	0 to 6	2.46	1.57	2	1 to 3	0 to 6
$-25 to $-5/animal	2.46	1.58	2	1 to 3	0 to 6	2.46	1.57	2	1 to 3	0 to 6
$-5 to $5/animal	2.46	1.57	2	1 to 3	0 to 6	2.45	1.57	2	1 to 3	0 to 6
$5 to $25/animal	2.46	1.57	2	1 to 3	0 to 6	2.46	1.57	2	1 to 3	0 to 6
> $25/animal	2.45	1.56	2	1 to 3	0 to 6	2.45	1.56	2	1 to 3	0 to 6

Supplemental Table 7. Frequency statistics of interest rates and their occurrence within net return difference categories for later fed endpoints (EPs) from a stochastic simulation model of feedlot steers marketed live or dressed with a premium and discount based grid[*,†]

	Interest rate, EP2			Interest rate, EP3			Interest rate, EP4		
Net return difference category, %	5%	7%	9%	5%	7%	9%	5%	7%	9%
Live sale basis									
< $-25/animal	32.2	33.2	34.6	31.9	33.4	34.7	31.8	33.3	35.0
$-25 to $-5/animal	33.5	33.2	33.4	33.6	33.1	33.3	33.3	33.4	33.3
$-5 to $5/animal	33.6	33.3	33.1	33.7	33.4	33.0	33.1	33.7	33.2
$5 to $25/animal	33.6	33.3	33.1	33.8	33.2	33.1	34.0	33.4	32.6
> $25/animal	34.5	33.6	31.9	35.0	33.4	31.6	35.5	33.3	31.1
Grid sale basis									
< $-25/animal	32.3	33.2	34.5	32.2	33.3	34.4	32.2	33.3	34.5
$-25 to $-5/animal	33.6	33.2	33.2	33.5	33.3	33.2	34.0	33.5	32.4
$-5 to $5/animal	33.8	33.1	33.1	33.9	33.3	32.8	34.8	33.4	31.8
$5 to $25/animal	33.3	33.3	33.4	33.8	33.4	32.9	34.6	33.7	31.7
> $25/animal	34.5	33.6	31.9	35.0	33.4	31.6	35.8	33.2	31.0

[*] Stochastic simulation models (n = 360,000 simulations) parameterized animal performance, carcass characteristics, health, as well as economic variables to evaluate differences in net returns when feeding steers to later endpoints under variable conditions

[†] Interest rates represented yearly fixed rates for operating loans, and were used to account for interest on 50% of feed as well as for accounting for opportunity cost when feeding to later EP

Supplemental Table 8. Descriptive statistics of carcass characteristic variables which were included in conditional random forest models, categorized by net return difference categories for later fed endpoints (EPs) from a stochastic simulation model of feedlot steers marketed dressed with a premium and discount based grid*,†

	Yield Grade 4 and 5 carcasses, %					Choice and Prime carcasses, %				
Net return difference category	Mean	SD	Med	IQR	95IPR	Mean	SD	Med	IQR	95IPR
Endpoint 2										
< $-25/animal	22.4	5.8	21.9	18.5 to 25.7	12.4 to 35.6	74.1	11.0	75.7	68.3 to 81.8	47.7 to 91.1
$-25 to $-5/animal	22.2	5.8	21.7	18.3 to 25.4	12.4 to 35.2	74.2	10.8	75.7	68.4 to 81.7	48.4 to 91.2
$-5 to $5/animal	22.0	5.7	21.5	18.2 to 25.3	12.2 to 35.0	74.2	10.8	75.7	68.3 to 81.8	48.7 to 91.0
$5 to $25/animal	22.0	5.7	21.5	18.2 to 25.3	12.3 to 34.7	74.1	10.8	75.6	68.2 to 81.7	48.4 to 91.0
> $25/animal	21.8	5.6	21.4	18.0 to 25.1	12.1 to 34.5	74.2	10.8	75.7	68.4 to 81.7	48.7 to 91.2
Endpoint 3										
< $-25/animal	27.9	6.7	27.4	23.4 to 31.8	16.1 to 42.5	77.8	10.0	79.3	72.7 to 84.7	53.3 to 92.7
$-25 to $-5/animal	27.6	6.6	27.1	23.2 to 31.4	16.0 to 42.2	77.7	10.0	79.3	72.6 to 84.6	53.1 to 92.6
$-5 to $5/animal	27.6	6.5	27.1	23.2 to 31.5	16.1 to 41.9	77.7	9.9	79.2	72.6 to 84.5	53.8 to 92.7
$5 to $25/animal	27.4	6.6	27.0	23.0 to 31.3	15.7 to 41.9	77.7	10.0	79.3	72.6 to 84.6	53.6 to 92.8
> $25/animal	27.3	6.5	26.8	22.9 to 31.2	15.7 to 41.7	77.8	9.9	79.3	72.6 to 84.6	53.9 to 92.7
Endpoint 4										
< $-25/animal	40.4	7.8	40.1	35.2 to 45.2	25.5 to 56.8	81.0	9.1	82.5	76.6 to 87.2	58.4 to 94.0
$-25 to $-5/animal	39.9	7.8	39.7	34.8 to 44.8	25.0 to 56.0	80.9	9.1	82.4	76.5 to 87.1	58.4 to 94.1
$-5 to $5/animal	39.8	7.9	39.7	34.5 to 44.8	24.5 to 56.1	81.0	8.9	82.4	76.4 to 87.1	59.6 to 93.9
$5 to $25/animal	39.8	7.9	39.5	34.6 to 44.6	24.7 to 56.0	80.9	9.1	82.4	76.5 to 87.0	58.7 to 94.1
> $25/animal	39.6	7.8	39.3	34.4 to 44.5	24.7 to 55.8	80.9	9.0	82.4	76.4 to 87.1	58.7 to 94.0

* Stochastic simulation models (n = 360,000 simulations) parameterized animal performance, carcass characteristics, health, as well as economic variables to evaluate differences in net returns when feeding steers to later endpoints under variable conditions

† Abbreviations: Med = median; IQR = interquartile range (25th to 75th percentile); 95IPR = 95th inter-percentile range (2.5th to 97.5th percentile);

Supplemental Table 8. Continued

	Heavyweight carcasses,[‡] %				
Net return difference category	Mean	SD	Med	IQR	95IPR
Endpoint 2					
< $-25/animal	21.8	6.6	21.3	17.0 to 26.0	10.6 to 36.3
$-25 to $-5/animal	21.3	6.6	20.7	16.6 to 25.4	10.3 to 35.8
$-5 to $5/animal	21.1	6.6	20.5	16.4 to 25.2	10.1 to 35.6
$5 to $25/animal	21.1	6.5	20.5	16.4 to 25.2	10.0 to 35.4
> $25/animal	21.0	6.5	20.4	16.3 to 25.0	10.1 to 35.4
Endpoint 3					
< $-25/animal	33.5	8.2	33.1	27.7 to 38.9	18.7 to 50.5
$-25 to $-5/animal	32.7	8.2	32.2	26.9 to 38.0	18.0 to 49.8
$-5 to $5/animal	32.7	8.1	32.3	26.9 to 38.0	18.2 to 49.7
$5 to $25/animal	32.6	8.1	32.2	26.8 to 38.0	18.0 to 49.6
> $25/animal	32.4	8.2	31.9	26.6 to 37.7	17.8 to 49.5
Endpoint 4					
< $-25/animal	47.0	9.0	46.9	40.7 to 53.1	29.7 to 64.7
$-25 to $-5/animal	46.0	9.1	45.8	39.7 to 52.2	28.6 to 63.8
$-5 to $5/animal	45.9	9.1	45.8	39.7 to 52.0	28.4 to 64.1
$5 to $25/animal	46.0	9.0	45.8	39.6 to 52.2	28.8 to 63.9
> $25/animal	45.8	9.1	45.6	39.5 to 51.9	28.4 to 63.8

[‡] Heavyweight carcasses were those weighing over 476 kg (1,050 lb)

Supplemental Table 9. Specified correlation coefficient matrices utilized for the parameterization of within-variable relationships during simulations*

Yield Grade correlation matrix†

	YG 5	YG 4	YG 3	YG 2
YG 5	1.00	0.97	0.96	0.93
YG 4	0.97	1.00	0.99	0.96
YG 3	0.96	0.99	1.00	0.97
YG 2	0.93	0.96	0.97	1.00

Quality Grade correlation matrix‡

	Prime	Choice	Select
Prime	1.00	0.97	0.86
Choice	0.97	1.00	0.89
Select	0.86	0.89	1.00

Fed cattle price correlation matrix§

	EP1 price	EP2 price	EP3 price	EP4 price
EP1 price	1.000	0.990	0.980	0.975
EP2 price	0.990	1.000	0.990	0.980
EP3 price	0.980	0.990	1.000	0.990
EP4 price	0.975	0.980	0.990	1.000

* Stochastic simulation models (n = 360,000 simulations) parameterized animal performance, carcass characteristics, health, as well as economic variables to evaluate differences in net returns when feeding steers to later endpoints under variable conditions

† Correlation matrix of USDA Yield Grades (YG) derived from the analysis of trial data using a generalized linear mixed model with a multinomial distribution and proportional odds framework; YG 1 was specified as the referent grade and was subsequently dropped from the model and therefore is not included in the matrix

‡ Correlation matrix of USDA Quality Grades (QG) derived from the analysis of trial data using a generalized linear mixed model with a multinomial distribution and proportional odds framework; Standard was specified as the referent grade and was subsequently dropped from the model and therefore is not included in the matrix

§ Correlation matrix of live fed cattle prices relative to each endpoint (EP) to account for changing prices when marketing on different weeks; correlations between weekly reported prices with two-week lags were analyzed to characterize the relationships and specify the matrix

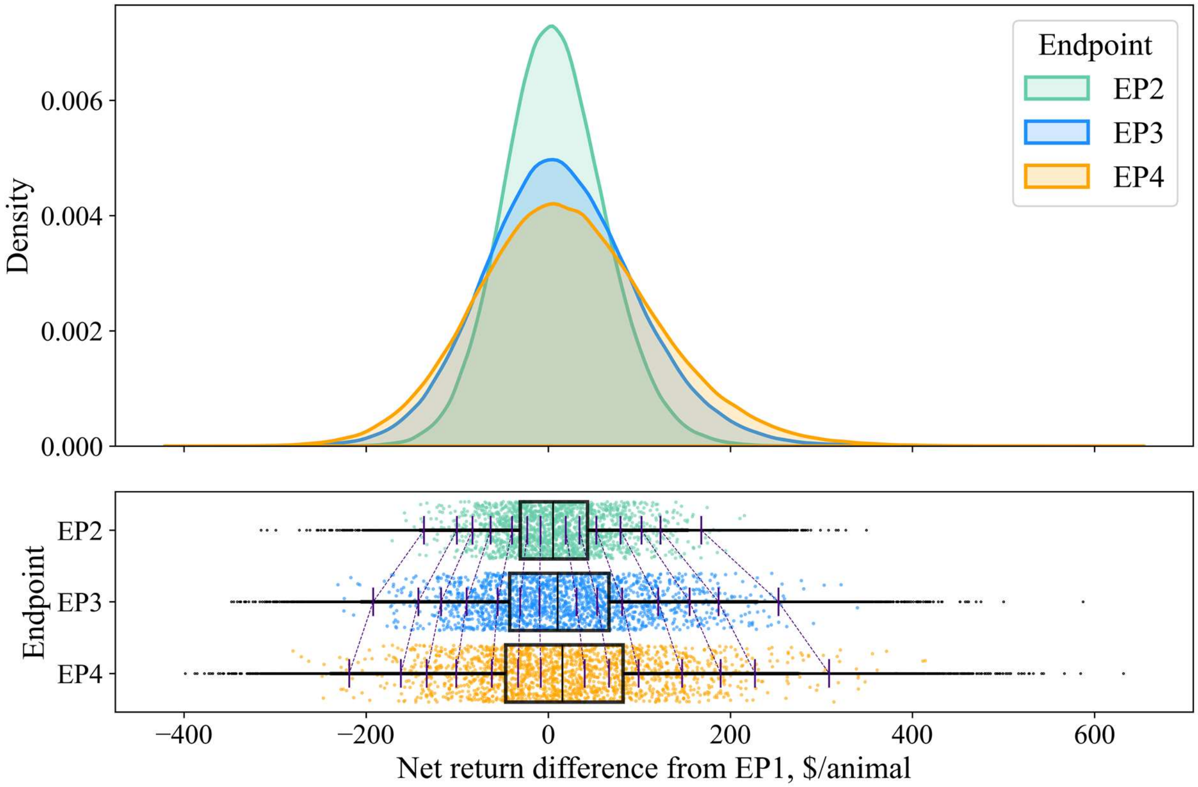

Supplemental Figure 1. Distributions of net return differences comparing later fed endpoints (EPs) to EP1 from a stochastic simulation model of beef feedlot steers marketed on a dressed (cash) basis (n = 360,000 simulations). Bars with connecting dotted lines are percentiles (from left to right: 0.5, 2.5, 5, 10, 20, 30, 40, 60, 70, 80, 90, 95, 97.5, and 99.5th percentiles, respectively). Raincloud plots show a random subsample of the data for visualization purposes, while boxplots and overlaid density plots use the full dataset.

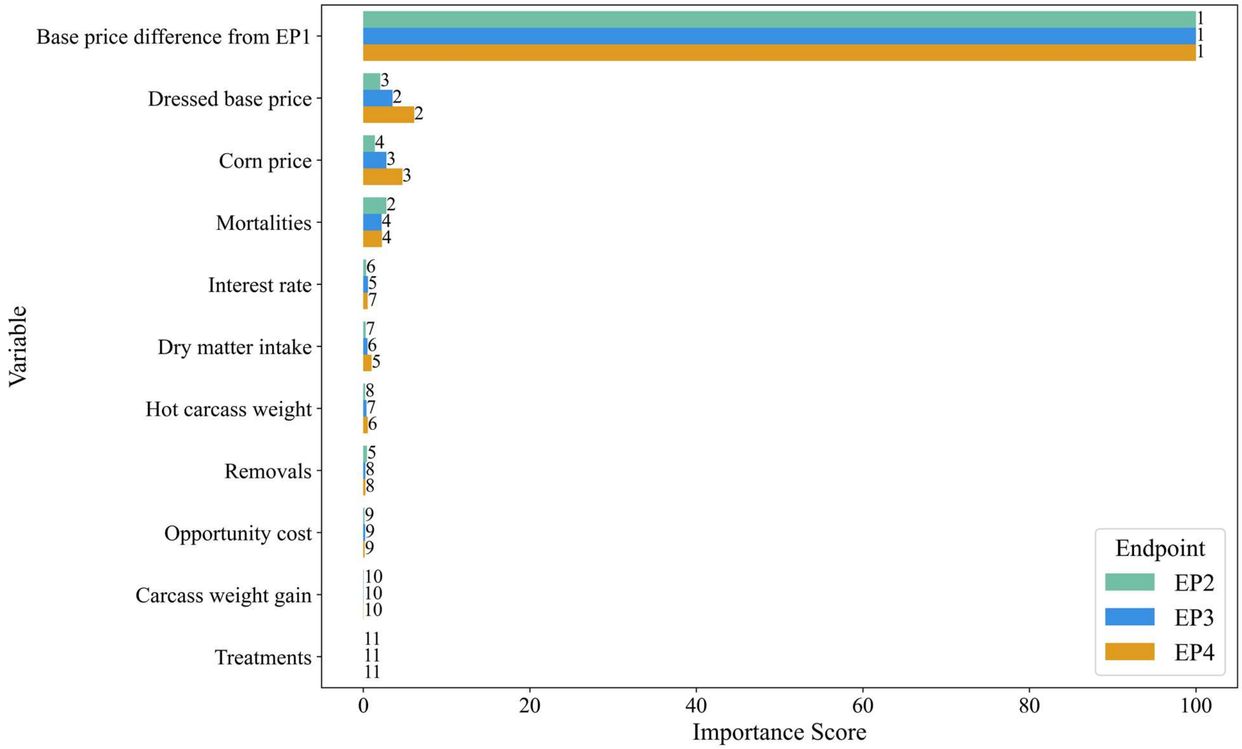

Supplemental Figure 2. Conditional variable importance scores from conditional random forest models to estimate the relative importance of the specified variables influencing net return differences of later fed endpoints (EP2, EP3, and EP4) compared to EP1 when marketing feedlot steers on a dressed (cash) sale basis in the stochastic simulation model. Data labels indicate the ranking from most to least important (1 to 11, respectively) within each EP.

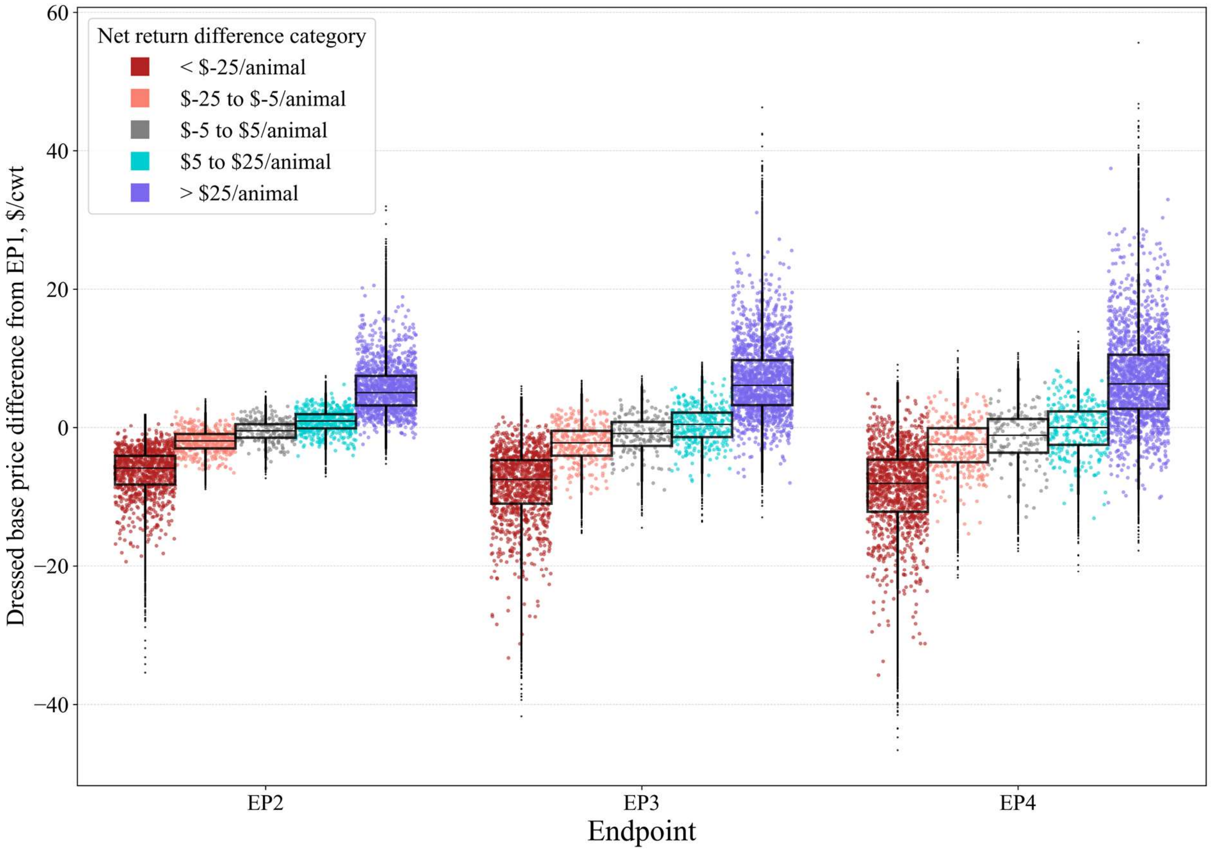

Supplemental Figure 3. Distribution of simulated dressed feed cattle base price differences compared to endpoint 1 (EP1), when categorized by net return differences (compared to EP1) when feeding steers to later EP and marketed on a dressed (cash) basis in a stochastic simulation model. Raincloud plots show a random subsample of the data for visualization purposes, while boxplots use the full dataset.
